# Development of a genome-editing CRISPR/Cas9 system in thermophilic fungal *Myceliophthora* species and its application to hyper-cellulase production strain engineering

**DOI:** 10.1186/s13068-016-0693-9

**Published:** 2017-01-03

**Authors:** Qian Liu, Ranran Gao, Jingen Li, Liangcai Lin, Junqi Zhao, Wenliang Sun, Chaoguang Tian

**Affiliations:** Key Laboratory of Systems Microbial Biotechnology, Tianjin Institute of Industrial Biotechnology, Chinese Academy of Sciences, Tianjin, 300308 China

**Keywords:** *Myceliophthora*, CRISPR/Cas9 system, Thermophilic fungi, Cellulases

## Abstract

**Background:**

Over the past 3 years, the CRISPR/Cas9 system has revolutionized the field of genome engineering. However, its application has not yet been validated in thermophilic fungi. *Myceliophthora thermophila*, an important thermophilic biomass-degrading fungus, has attracted industrial interest for the production of efficient thermostable enzymes. Genetic manipulation of *Myceliophthora* is crucial for metabolic engineering and to unravel the mechanism of lignocellulose deconstruction. The lack of a powerful, versatile genome-editing tool has impeded the broader exploitation of *M. thermophila* in biotechnology.

**Results:**

In this study, a CRISPR/Cas9 system for efficient multiplexed genome engineering was successfully developed in the thermophilic species *M. thermophila* and *M. heterothallica*. This CRISPR/Cas9 system could efficiently mutate the imported *amdS* gene in the genome via NHEJ-mediated events. As a proof of principle, the genes of the cellulase production pathway, including *cre*-*1*, *res*-*1*, *gh1*-*1,* and *alp*-*1*, were chosen as editing targets. Simultaneous multigene disruptions of up to four of these different loci were accomplished with neomycin selection marker integration via a single transformation using the CRISPR/Cas9 system. Using this genome-engineering tool, multiple strains exhibiting pronounced hyper-cellulase production were generated, in which the extracellular secreted protein and lignocellulase activities were significantly increased (up to 5- and 13-fold, respectively) compared with the parental strain.

**Conclusions:**

A genome-wide engineering system for thermophilic fungi was established based on CRISPR/Cas9. Successful expansion of this system without modification to *M. heterothallica* indicates it has wide adaptability and flexibility for use in other *Myceliophthora* species. This system could greatly accelerate strain engineering of thermophilic fungi for production of industrial enzymes, such as cellulases as shown in this study and possibly bio-based fuels and chemicals in the future.

**Electronic supplementary material:**

The online version of this article (doi:10.1186/s13068-016-0693-9) contains supplementary material, which is available to authorized users.

## Background

Lignocellulosic biomass is an abundant renewable, conventional energy source for many industrial applications [1, 2]. Through their secretion of large amounts of lignocellulolytic enzymes to break down lignocellulose to fermentable sugar, saprobic ascomycete and basidiomycete filamentous fungi are the main decomposers of plant biomass [3]. The thermophilic filamentous fungus *Myceliophthora thermophila* is exceptionally attractive for biotechnological applications, with multiple characteristics of industrial interest, such as high-temperature fermentation, substantial protein secretion capacity, and fast growth on cellulose [4–8]. Consequently, *M. thermophila* has been developed into a platform for industrial enzyme production (C1 strain) [9] and also has the biotechnological potential to be a cell factory to produce chemicals and biofuels from biomass polysaccharides [7]. The genome of the *M. thermophila* wild-type (WT) strain ATCC 42464 has been completely sequenced and annotated [10], thereby allowing systematic examination and identification of lignocellulolytic enzymes cultured in defined plant-derived biomass through transcriptome and exoproteome approaches [5, 11].

Recently, a high-efficiency *Agrobacterium tumefaciens*-mediated transformation system was developed by our group for the sequenced *M. thermophila* strain ATCC 42464 [12]. Using this technique, a *ku70* deletion strain was constructed. The homologous recombination (HR) rate was improved approximately threefold in the Δ*ku70* mutant, thereby facilitating the high-throughput generation of single-gene deletions of *M. thermophila*. However, although classical gene deletion approaches using homologous integration with flanking regions longer than 1000-bp have been applied in filamentous fungi [13], they are technically complicated and time intensive, particularly for multiple gene engineering. Because no sexual cycle has yet been found in *M. thermophila*, multiple gene disruption cannot be achieved by sexual crossing. Thus, the lack of a powerful and versatile genome-editing tool for *M. thermophila* impedes wider biotechnological exploitation of this fungus.

The type-II prokaryotic clustered regularly interspaced short palindromic repeats (CRISPR)/Cas adaptive immune system has recently been engineered into a powerful genome-editing system and has been broadly applied across species [14–24]. Briefly, this system consists of a Cas9 endonuclease and a synthetic single chimeric guide RNA (sgRNA), the latter a fusion of CRISPR RNA (crRNA) and trans-activating crRNA (tracrRNA). In the commonly used form of Cas9, the sgRNA consists of a 20-bp protospacer sequence recognizing the target site by base pairing and a downstream gRNA scaffold sequence. The target DNA must contain the 20-bp genomic target sequences followed by the requisite 3-bp protospacer adjacent motif (PAM) 5′-NGG. The mature gRNA guides Cas9 to specific 20-nucleotide genomic loci, and Cas9 then introduces a double-strand break (DSB) at the target DNA upstream of the PAM. A DSB generated by Cas9 can be repaired by nonhomologous end joining (NHEJ) to directly carry out indel mutagenesis; alternatively, homology-directed repair (HDR) can be accomplished if a DNA repair template (donor DNA) is simultaneously provided with the sgRNA. Besides introducing DSBs, recent studies have shown that a catalytically inactive dCas9 protein lacking endonuclease activity can efficiently generate a RNA-guided regulation platform for sequence-specific control of gene repression (CRISPRi) and activation (CRISPRa) with minimal off-target effects [25–27].

The functional CRISPR/Cas9 system for gene editing has been successfully developed in yeasts and some fungi [28–40], including industrial filamentous fungi *Aspergillus stains* [33, 40], *Trichoderma reesei* [34] and *Penicillium chrysogenum* [36], and plant pathogenic fungi *A. fumigatus* [35, 38], *Magnaporthe oryzae* [37], and *Ustilago maydis* [39]. For instance, by using the CRISPR/Cas9 technology in *T. reesei* [34], simultaneous engineering of multiple genes was efficiently achieved through co-transformation with in vitro synthesized gRNAs and donor DNA even using short 200-bp homology arms, providing an applicable and promising approach to other lignocellulose degrading filamentous fungi. In *A. fumigatus* [35], the MMEJ-CRISPR system consisted of Cas9, and in vivo synthesized sgRNA under control of the *A. fumigatus* U6 snRNA promoter was sufficient to introduce 95–100% precise integration via even very short 35-bp homologous arms, indicating that this system can function as a powerful and versatile genome-engineering tool for genetic investigation in clinical *Aspergillus* isolates. Remarkably, it recently has been reported that the Di-CRISPR platform can efficiently integrate 18 copies of a combined xylose utilization and (*R*,*R*)-2,3-butanediol (BDO) production pathway (24 kb) in the genome of *Saccharomyces cerevisiae* in a single step and generate a strain capable of producing BDO directly from xylose [31], envisioning that CRISPR technologies possess great potential of industrial application in metabolic engineering.

The development and application of the CRISPR/Cas9 system has not yet been reported in thermophilic fungi however. In this study, a CRISPR/Cas9 system was developed and successfully applied for genome engineering of up to four different gene loci simultaneously in the thermophilic fungus of industrial interest *M. thermophila*. Using this system, multiple strains with significantly increased lignocellulase production were generated. Successful targeted gene deletion was also demonstrated in *M. heterothallica* [41–43], suggesting that the system developed here could be used in other thermophilic fungi. This new tool should accelerate the engineering of thermophilic fungi for industrial biotechnological production of enzymes and chemicals.

## Results

### Expression of constitutive Cas9 in *Myceliophthora thermophila*

**Fig. 1:**
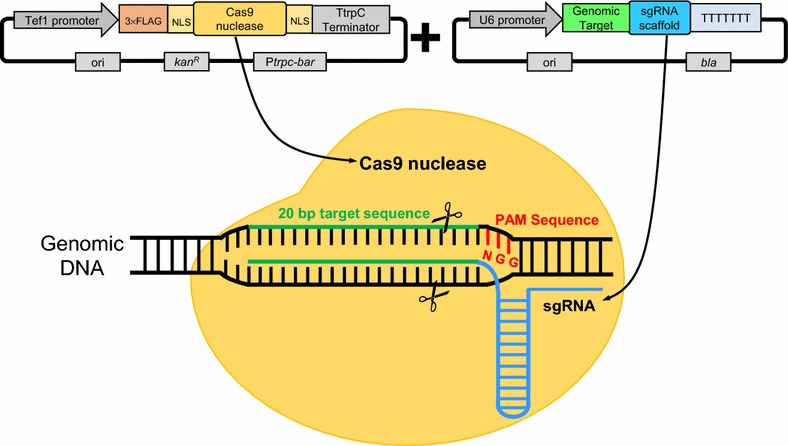
Schematic representation of the CRISPR/Cas9 two-plasmid system in this study

**Fig. 2:**
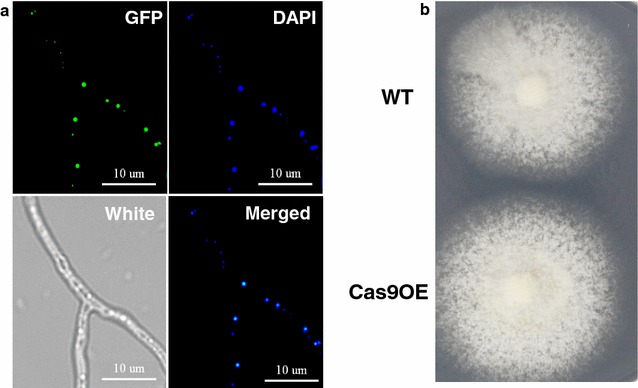
Phenotypic analysis of constitutive Cas9-expressing strains. **a** Fluorescence microscopic assessment of Cas9-gfp localization in *M. thermophila*. The nuclei were stained with 4′,6-diamidino-2-phenylindole. Each *scale bar* represents 10 µm. **b** Colony growth and sporulation of Cas9OE and wild-type (WT) strains on minimal medium plates after 4 days of culture

To develop an efficient CRISPR/Cas9 system (Fig. 1) for thermophilic fungi, an expression plasmid containing the P*tef1*-*Cas9*-T*tprC* cassette and a *bar* selection marker was constructed. This expression vector was introduced into the WT strain of *M. thermophila* ATCC 42464. To check Cas9 expression and localization, a second plasmid with the enhanced green fluorescent protein (e*GFP*) gene fused to *Cas9* was then constructed and transformed into the strain ATCC 42464. The positive transformants from each construct were selected and named Cas9OE and Cas9-gfp accordingly. Microscopic analysis clearly demonstrated that Cas9 correctly localized to the nucleus of *M. thermophila* (Fig. 2a).

To determine whether the constitutive expression of Cas9 nuclease alone had an effect on the growth of *M. thermophila*, the growth, secreted protein production, lignocellulosic enzyme activity, and mycelial dry weight of the Cas9OE strain were examined after three consecutive subcultures in parallel with those of the WT (ATCC 42464). As illustrated in Fig. 2b, the Cas9OE strain and the WT exhibited similar colony growth when grown on a sucrose carbon source. No significant difference (Tukey’s HSD, *p* = 0.89) in secreted protein (Additional file 1: Fig. S1A, B) or lignocellulase activity (Additional file 1: Fig. S1C–E) was observed after 3 or 4 days of fermentation on 2% Avicel liquid medium. The Cas9OE strains showed an identical dry weight when grown on sucrose and Avicel (Additional file 1: Fig. S1F). Taken together, all of these results indicated that constitutive expression of Cas9 nuclease did not alter the growth, sporulation, or cellulolytic production of the ATCC 42464 strain of *M. thermophila*.

### CRISPR/Cas9 system-directed gene mutagenesis without donor DNA in *Myceliophthora thermophila*

Because efficient expression of sgRNA requires a suitable promoter, a search for the native promoter of putative U6 snRNA genes in the genome of *M. thermophila* ATCC 42464 was conducted using bioinformatics tools. The putative RNA polymerase III U6 snRNA genes (*U6*) were located (http://genome.jgi.doe.gov) and a region approximately 500 bp upstream of the *U6* gene was chosen as a trial promoter for expression of sgRNA (Fig. 1).

**Fig. 3:**
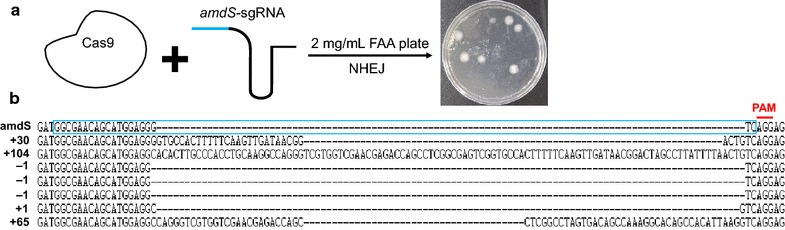
Introduction of directed mutations into the *amdS* gene in *M. thermophila* by sgRNA-guided Cas9. **a** Schematic illustration of *amdS* mutagenesis by the CRISPR/Cas9 system. The M1 strain carrying the *amdS* gene was co-transformed with U6p-amdS-sgRNA and Cas9 cassettes, and then positive transformants were selected on medium plates containing 2 mg/mL FAA. **b** Sequence alignment of the *amdS* target locus from FAA-resistant transformants. The wild-type target sequence of *amdS* is framed in* blue*.* Red letters* depict the protospacer adjacent motif (PAM)

To investigate whether the constructed CRISPR/Cas9 system (Fig. 1) could perform gene editing in *M. thermophila*, a rapid testing system was designed based on *amdS/*fluoroacetamide (FAA) selection. This system was chosen because *M. thermophila* WT strains do not grow on acetamide as a nitrogen source and are resistant to certain FAAs. The transformants carrying the *amdS* gene were sensitive on FAA medium; thus, colonies harboring inactivated *amdS* could be isolated on FAA medium, thereby facilitating counter selection. Therefore, the *amdS* gene of *A. nidulans* was first artificially introduced into the *M. thermophila* WT genome through *Agrobacterium*-mediated transformation [12]. The transformant carrying the functional *amdS* gene was named M1. An sgRNA sequence containing a 20-nucleotide target (Table 1) of the *amdS* gene was designed (U6p-*amdS*-sgRNA) to perform the *amdS* gene editing. The transient PCR product of U6p-*amdS*-sgRNA was co-transformed with a Cas9 expression cassette into protoplasts of the recipient strain M1 (containing the *amdS* gene and FAA-sensitive) (Fig. 3a). Seven FAA-resistant colonies were obtained and their *amdS* gene mutations were verified by sequencing. The seven mutants displayed frame-shift mutations upstream of the PAM site (Fig. 3b) involving either a single-nucleotide insertion/deletion or large insertions of 30, 65, or 104 nucleotides (the 104-nucleotide insertion mutant was named the M2 stain, and was used as a host strain for subsequent engineering). In a control experiment in which transformation was conducted using the Cas9 plasmid only, no colonies were observed on FAA-containing medium. Taken together, these data demonstrated that the CRISPR/Cas9 system could mutate the target gene in the *M. thermophila* genome via NHEJ-mediated events during DSB repair.

**Table 1:** List of guide and protospacer adjacent motif (PAM) sequences of each target locus used in this study

Target locus	Guide sequence	PAM
*amdS*	GGCGAACAGCATGGAGGGTC	AGG
*cre*-*1*	GCAACGCGCAAAGTCTGCAG	TGG
*res*-*1*	GCCCTATGAGCCCTCGTACC	CGG
*gh1*-*1*	GACACATTCTGCGCCATCCC	CGG
*alp*-*1*	GTCTACCGCGGCAAGTTCAG	GGG

### CRISPR/Cas9 system-stimulated gene editing with donor DNA in *Myceliophthora thermophila* and *M. heterothallica*

CRISPR/Cas9 system-mediated HDR has been shown to work efficiently in many organisms, including several mesophilic fungi [33–40]. To determine whether the CRISPR/Cas9 system could also operate via HDR on target gene loci in *M. thermophila*, a transient single-sgRNA PCR cassette was transformed into ATCC 42464 WT protoplasts together with a Cas9 expression cassette and a donor DNA PCR product composed of the G418-resistance cassette *PtrpC*-*neo* and 5′ and 3′ homologous arms.

**Fig. 4:**
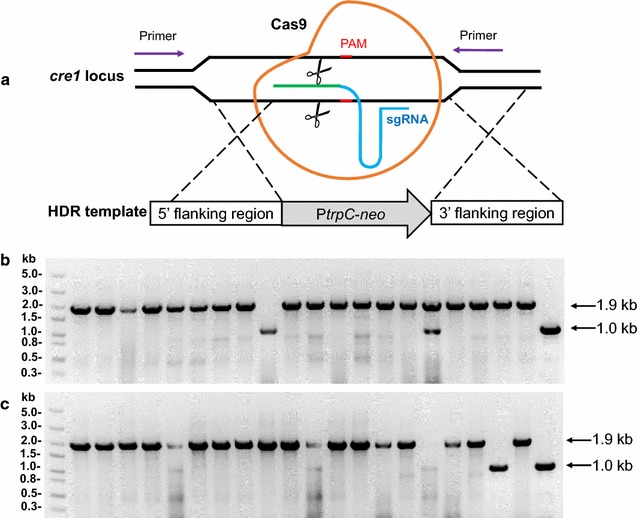
Verification of *cre*-*1* deletion in selected transformants. **a** Schematic of homologous recombination (HR) of the target gene *cre*-*1* mediated by Cas9, gRNA and donor DNA. (**b-c**) PCR analysis of selected transformants of *M. thermophila* **b** and *M. heterothallica* **c** with one primer (cre1-out-F) located upstream of the 5′ flanking region of the genomic DNA and the other (cre1-in-R) located in the 3′ flanking region of the genomic DNA. The expected length of disrupted transformants was 1.9 kb, while that of the host strain, used as a negative control, was 1.0 kb (rightmost lane)

As a proof of principle, the carbon catabolite repression (CCR) transcription factor *cre*-*1* [44] was chosen as a target locus for editing (Fig. 4a). The protospacer sequence targeted to *cre*-*1* is shown in Table 1. On the basis of genomic PCR analysis using specific primer sets (Fig. 4a), the selected marker cassettes were confirmed to be correctly inserted into the desired locus (Δ*Mtcre*-*1*) in 19 out of 20 randomly picked transformants (Fig. 4b). The homologous recombination (HR) efficiencies for all tested transformants are summarized in Table 2. Compared with transformation with donor DNA alone (20%), the HR frequency of gene replacement was as high as 95% after co-transformation using the CRISPR-Cas9 system and HR donor DNA (Table 2; Fig. 4b; Additional file 2: Fig. S2). The HR rates were low (15%) when Cas9 or sgRNA alone was used for the transformation (Table 2; Additional file 2: Fig. S2).

**Table 2:** CRISPR/Cas9-mediated homologous recombination (HR) efficiency of simultaneous disruption of one to four gene loci

Host strain	Target locus	Elements in co-transformation	No. of analyzed transformants	No. of HR transformants	HR efficiency (%)
ATCC 42464	*cre*-*1*	Donor-*cre1*	20	4	20
ATCC 42464	*cre*-*1*	*Cas9* + donor-*cre1*	20	3	15
ATCC 42464	*cre*-*1*	*cre1*-sgRNA + donor-*cre1*	20	3	15
ATCC 42464	*cre*-*1*	*Cas9* + *cre1*-sgRNA + donor-*cre1*	20	19	95
CBS 203.75	*cre*-*1*	Donor-*cre1*	20	4	20
CBS 203.75	*cre*-*1*	*Cas9* + donor-*cre1*	20	3	15
CBS 203.75	*cre*-*1*	*cre1*-sgRNA + donor-*cre1*	20	4	20
CBS 203.75	*cre*-*1*	*Cas9* + *cre1*-sgRNA + donor-*cre1*	20	18	90
M2 strain	*res*-*1*, *gh1*-*1*	Two sets of sgRNA and donor DNA of *res*-*1* and *gh1*-*1*	23	15	65
M2 strain	*cre*-*1*, *gh1*-*1*	Two sets of sgRNA and donor DNA of *cre*-*1* and *gh1*-*1*	23	14	61
M2 strain	*cre*-*1*, *res*-*1*	Two sets of sgRNA and donor DNA of *cre*-*1* and *res*-*1*	23	16	70
M2 strain	*cre*-*1*, *res*-*1*, *gh1*-*1*	Three sets of sgRNA and donor DNA of *cre*-*1*, *res*-*1* and *gh1*-*1*	23	7	30
M2 strain	*cre*-*1*, *res*-*1*, *gh1*-*1*, *alp*-*1*	Four sets of sgRNA and donor DNA of *cre*-*1*, *res*-*1*, *gh1*-*1* and *alp*-*1*	23	5	22

To evaluate the possibility of broader application of the above-described CRISPR/Cas9 system to other thermophilic fungi, it was used to perform genome editing in *M. heterothallica*, also with *cre*-*1* as the target gene. The Cas9, U6p-*cre1*-sgRNA, and donor-*cre1* constructs were co-transformed into *M. heterothallica* strain CBS203. Using both G418 and phosphinothricin selection, 20 transformants were chosen randomly and validated by PCR. As illustrated in Table 2, Fig. 4c, this system functioned well in CBS203.75, with the G418 marker cassette successfully integrated into the *cre*-*1* locus of 18 transformants with 90% HR frequency. In the control experiments, only 15–20% disruption efficiencies were obtained when Cas9 or sgRNA was used for the transformation (Table 2; Additional file 2: Fig. S2).

### Deletion of *cre*-*1* led to increased cellulase production

**Fig. 5:**
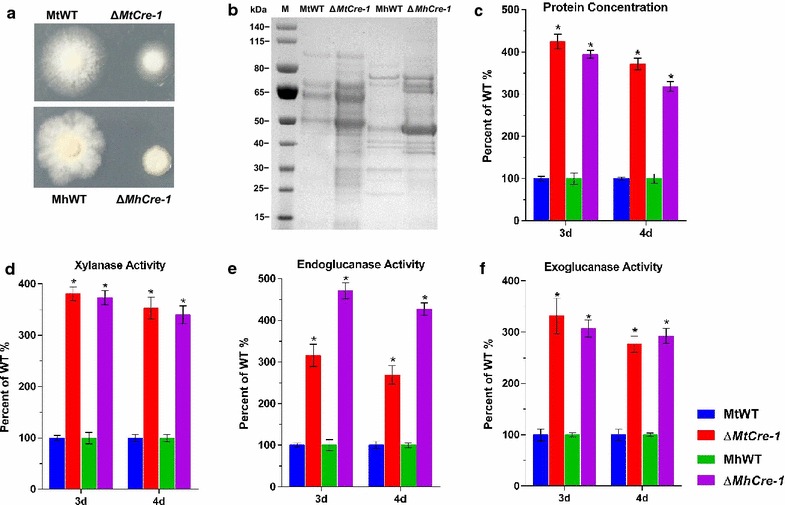
Phenotypic analysis of Δcre-1 and wild-type strains. **a** Colonies of Δ*cre*-*1* (Δ*Mtcre*-*1* and Δ*Mhcre*-*1*) and wild-type strains (MtWT and MhWT) of *M. thermophila* and *M. heterothallica* on minimal medium plates after 2 days. **b** Sodium dodecylsulfate-polyacrylamide gel electrophoresis of secreted protein of Δ*cre*-*1* and wild-type strains after 4 days culture in 2% Avicel medium. **c**–**f** Assays for protein concentration and xylanase, endoglucanase, and exoglucanase activities of Δ*cre*-*1* and wild-type strains in 2% Avicel inducing medium after 3 and 4 days culture. *Bars* marked by *asterisks* in each group differ significantly from unmarked ones (Tukey’s HSD, *p* < 0.001). *Error bars* represent SD from three replicates

Similar to the morphological alterations observed in *cre1* deletion mutants of *T. reesei*, *Neurospora crassa*, and *P. oxalicum* [44–49], the *M. thermophila* Δ*Mtcre*-*1* mutant exhibited slower, denser growth on sucrose than the WT ATCC 42464 (Fig. 5a). The effect of the *cre*-*1* deletion on cellulase and hemicellulase production was also investigated. Compared with the WT strain, the Δ*Mtcre*-*1* mutant secreted 3.7-fold higher amounts of extracellular protein into the Avicel medium after 4 days of cultivation (Fig. 5b, c). Xylanase, endoglucanase, and exoglucanase activities in Δ*Mtcre*-*1* were all increased approximately threefold compared with the WT strain in 2% Avicel (Fig. 5d–f). Similar to the phenotype of Δ*Mtcre*-*1*, the *M. heterothallica* Δ*Mhcre*-*1* mutant also formed smaller, denser colonies relative to the WT strain CBS203 (Fig. 5a). When grown on medium containing 2% Avicel as the sole carbon source, the Δ*Mhcre*-*1* mutant displayed a pronounced increase in secreted protein levels and lignocellulase activities (Fig. 5b–f) compared with the WT strain CBS203 (Tukey’s HSD, *p* < 0.001).

### CRISPR/Cas9-mediated simultaneous multiplex-locus genome editing in *Myceliophthora thermophila*

Because the CRISPR/Cas9 system functioned very efficiently for single-gene (e.g., *cre*-*1* locus) editing in *M. thermophila*, multiple gene disruption through a single transformation was investigated. In addition to *cre*-*1*, two genes involved in cellulase production were chosen as targets: an endoplasmic reticulum stress regulator *res*-*1* homolog [50] and the *gh1*-*1* gene encoding β-glucosidase [10, 49, 51–53]. M2, a strain with constitutive Cas9 expression and inactive *amdS* (through a large insertion of 104 nucleotides) that was derived from M1 (a strain carrying the *amdS* gene under the WT background, see Fig. 3), was used as the recipient strain in further experiments. First, three combinations of two-gene editing were performed (*cre1* + *res1*, *cre1* + *gh1-1*, and *res1* + *gh1-1*; Table 2). The sgRNA and donor DNA of each of the two genes were co-transformed in a one-step transformation. A total of 23 transformants were randomly picked on selective plates containing G418 and phosphinothricin. All 23 randomly chosen transformants were then checked for both genes by diagnostic PCR. Using the CRISPR/Cas9 editing system, the efficiency of simultaneous disruption of the two genes was approximately 61–70% (Additional file 3: Fig. S3; Table 2). No obvious preference for a particular tested locus was observed in regard to disruption efficiency. A total of 10 Δ*cre1*Δ*gh1*-*1*, 11 Δ*cre1*Δ*res1*, and 11 Δ*gh1*-*1*Δ*res1* homokaryotic double deletions were obtained (Additional file 3: Fig. S3).

Next, simultaneous deletion of all three loci (*cre1*, *res1*, and *gh1*-*1*) was conducted. Using the sgRNA and donor DNA sequences described above, all three sgRNA/donor DNA gene disruption sets were co-transformed together in the same molar amounts into the M2 strain. After 3 days of culture in selective medium, 23 transformants were randomly picked and subjected to PCR analysis. Seven transformants (homokaryon + heterokaryon) showed homologous recombination in the *cre1*, *res1*, and *gh1*-*1* gene loci, and the efficiency of simultaneous triple recombination was about 30% (Additional file 4: Fig. S4; Table 2). Via one transformation, in particular, five of seven possible combinations of mutant genotypes were obtained, including the single-gene mutant Δ*cre*-*1*, all three double-mutant genotypes (Δ*cre1*Δ*gh1*-*1*, Δ*cre1*Δ*res1*, and Δ*gh1*-*1*Δ*res1*) and the triple mutant Δ*cre1*Δ*gh1*-*1*Δ*res1*, with only two single-gene mutants (Δ*res1* and Δ*gh1*-*1*) missing (Additional file 4: Fig. S4).

Finally, simultaneous four gene disruption mediated by the CRISPR/Cas9 system was performed. In addition to *cre1*, *res1*, and *gh1*-*1*, the gene *alp*-*1* [9], encoding alkaline protease, was chosen as the fourth deletion target. The experiment was performed as described above for three-gene deletion. Among 23 selected transformants, five clones (homokaryon + heterokaryon, 22%) were identified with all four genes disrupted, including three with homokaryotic quadruple deletions (Δ*c*Δ*g*Δ*r*Δ*a*) (Additional file 5: Fig. S5). The copy number of the integrated *neo* marker in these transformants was determined by real-time quantitative PCR (RT-qPCR). Generally speaking, the copy number of the integrated marker was in good accordance with the number of target genes, which indicated that additional random integration of the marker did not happen often in this fungus (Additional file 6: Fig. S6).

### Acquisition of multiple hyper-cellulase production strains through multiplex-locus genome engineering with the CRISPR/Cas9 technique in thermophilic fungi

**Fig. 6:**
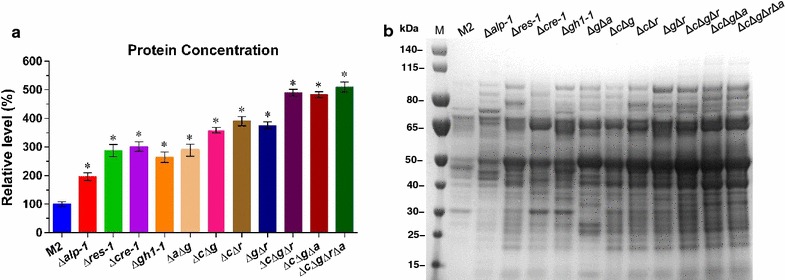
Secretome analysis of all disrupted mutants and the parental strain (M2). **a** Secreted protein production of all strains after 6 days cultivation in 2% Avicel inducing medium plus 0.5% peptone. **b** Sodium dodecylsulfate-polyacrylamide gel electrophoresis of secreted protein in 6-day Avicel cultures.* Bars* marked by* asterisks* in each group differ significantly from unmarked ones (Tukey’s HSD, *p* < 0.001). *Error bars* represent SD from three replicates

**Fig. 7:**
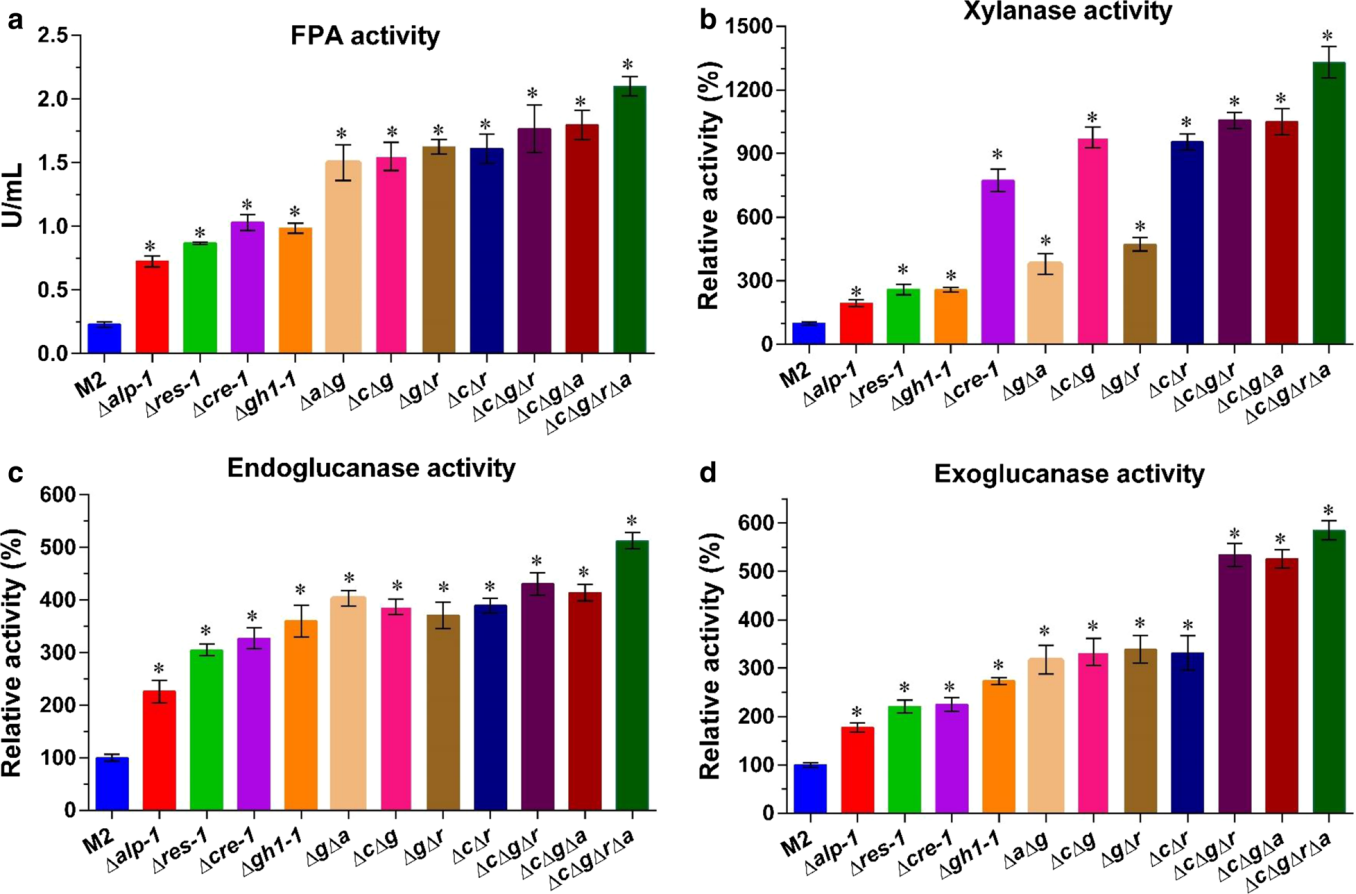
Altered cellulolytic phenotypes of all disrupted mutants vs. the parental strain (M2). Assessments of FPA (**a**), xylanase (**b**), endoglucanase (**c**), and exoglucanase (**d**) activities of all strains in 2% Avicel inducing medium plus 0.5% peptone after 6 days culture. *Bars* marked by *asterisks* in each group differ significantly from unmarked ones (Tukey’s HSD, *p* < 0.001). *Error bars* represent SD from three replicates

As described above, four genes involved in the cellulase production pathway in *M. thermophila* were engineered using the CRISPR/Cas9 system and a total of 11 mutants (including single to quadruple gene deletion mutants) were obtained through multiple operations. Secreted protein levels and cellulolytic enzyme activities were measured in these 11 mutants (Figs. 6, 7). As shown in Fig. 6, all 11 mutants exhibited higher secreted protein production than the parental strain M2. Several of these mutants, including all obtained double, triple, and quadruple deletion mutants, produced secreted protein at levels threefold higher than that of the original strain. Of particular note, the secretome of the quadruple deletion mutant Δ*c*Δ*g*Δ*r*Δ*a* was approximately fivefold larger than that of the parental strain.

Consistent with this increased secreted protein production, lignocellulase activities in the mutants were markedly higher than in the original strain (Fig. 7). The filter paper activity (FPA, representing overall cellulase activity) and xylanase, endoglucanase, and exoglucanase activities in all mutants were significantly increased relative to the parental strain (Tukey’s HSD, *p* < 0.001) (Fig. 7). It was exciting to observe that FPA, xylanase, endoglucanase, and exoglucanase activities in three strains, Δ*c*Δ*g*Δ*r*, Δ*c*Δ*g*Δ*a*, and Δ*c*Δ*g*Δ*r*Δ*a*, were, respectively, 7.7- to 9.1-, 10.5- to 13.3-, 4.1- to 5.1-, and 5.3- to 5.8-fold higher than in the parental strain.

## Discussion

In this study, a CRISPR/Cas9 system was successfully developed that could disrupt single or multiple genes in *M. thermophila* and another *Myceliophthora* species, *M. heterothallica*. Previously, the U6 and other RNAP III promoters have commonly been used for expressing sgRNA during the development of CRISPR/Cas9-based genome-editing tools in various organisms, including fungi such as *A. fumigatus* [35], *P. chrysogenum* [36], *M. oryzae* [37], and *U. maydis* [39]. However, these promoters are ill defined in some filamentous fungi such as *T. reesei*, in which the sgRNA was synthesized in vitro [34]. To minimize the number of transformation steps necessary to target *M. thermophila* genes, the U6 promoter of *M. thermophila* was identified here and used to drive the expression of sgRNAs in genome editing. Compared with classical gene deletion approaches such as *A. tumefaciens*-mediated transformation [12] and gene knockdown method by using the RNA interference [44], genome engineering in *M. thermophila* using the CRISPR/Cas9 strategy is much faster and easier. For example, the disruption efficiency of a single gene (*cre*-*1*) was as high as 90% using the CRISPR/Cas9 technology (Fig. 4), whereas the deletion frequency was only ~20% using the classical gene deletion method for the same gene (Additional file 2: Fig. S2). There has been great progress in the development of CRISPR/Cas9-based genome-editing tools in cellulolytic fungi; using in vitro synthesized sgRNA, double (HR frequency: 16−45%) and triple (4.2%) gene disruption was achieved through one transformation using the CRISPR/Cas9 system in *T. reesei* [34]. Compared with the in vitro synthesized sgRNA in the *T. reesei* system, the recombination efficiency was higher in *M. thermophila* using the CRISPR/Cas9 system developed in this study (~60, ~30, and ~20% for double, triple, and quadruple deletion), in which the sgRNA was transcribed in vivo by the U6 promoter from itself (Table 2).

In cellulolytic fungi, lignocellulolytic enzyme production is regulated at both the transcriptional [54] and post-translational levels [50]. Genetically engineering the regulatory network at both levels represents an efficient and promising strategy for improving cellulase production in filamentous fungi. For instance, mis-expression of the transcription factors *cre1* [45–49] and *res1* [50], and the major protease gene *alp*-*1* [9] has resulted in significantly improved lignocellulolytic enzyme production. In the present study, the four genes (*cre1*, *res1*, *gh1*-*1*, and *alp*-*1*) involved in the cellulase production pathway in *M. thermophila* were simultaneously engineered by a one-step transformation, using the CRISPR/Cas9 system developed here. Compared with classical deletion methods, which generally yield a single mutant genotype per transformation, this time-saving CRISPR/Cas9 system can generate multiple mutant genotypes with different combinations of disrupted genes and thus provides additional opportunities to obtain desired mutant strains of industrial interest. For example, in present study, for the three-gene deletion assay (*cre1, gh1*-*1*, *res1*), besides the triple mutant (Δ*cre1*Δ*alp1*Δ*res1*), a total 5 of 7 possible mutant genotypes from single to triple mutants were obtained. Only two single-gene mutants (Δ*res1* and Δ*gh1*-*1*) were missing, which might have been because only a limited number of transformants (23 for each transformation) were picked; all the gene deletion combinations could potentially be obtained by screening more transformants. Thus, this method paves the way to get more desirable mutants by straightforward screening of colonies from a single transformation.

The deletion of *cre1* in *M. thermophila* and *M. heterothallica* led to obvious increases in the cellulase secretome and activities, which suggested that the mechanism of cellulase repression mediated by *cre*-*1* is conserved in the thermophilic fungus *Myceliophthora* and the mesophilic fungi *N. crassa*, *T. reesei,* and *P. oxalicum* [44–49]. As expected, the triple and quadruple deletion strains Δ*c*Δ*g*Δ*r*, Δ*c*Δ*g*Δ*a*, and Δ*c*Δ*g*Δ*r*Δ*a* displayed approximately fivefold higher cellulase production and 5.1–13.3-fold higher cellulolytic enzyme activities than the parent strains. The cellulase production levels by the strains developed here from one transformation are comparable to those of strains engineered by time-consuming methods in the well-known cellulase production species *T. reesei* [55] and *P. oxalicum* [53], suggesting that strain engineering for cellulase production in *M. thermophila* could be improved significantly within a short time through the CRISPR/Cas9 system developed here. Additionally, this CRISPR/Cas9 system can be used without modification in other *Myceliophthora* species such as *M. heterothallica* [41–43], indicating it could be potentially used in many thermophilic fungi. This broad applicability provides an opportunity for deep investigation of these fungi, some of which possess interesting features such as a sexual cycle, both for basic research and for possible novel host development for future industrial biotechnological use.

## Conclusions

In this study, an efficient CRISPR/Cas9 system for genome editing was successfully developed in the thermophilic species *M. thermophila* and *M. heterothallica*. Using this system, up to four genes involved in the cellulase production pathway were simultaneously deleted in a one-step transformation. A strain with more than fivefold higher lignocellulase production was obtained after a single engineering cycle. The CRISPR/Cas9 system developed in this study should accelerate genome-wide metabolic engineering of thermophilic fungal *Myceliophthora* species for the production of lignocellulases and bio-based fuels and chemicals.

## Methods

### Strains and growth conditions

For vector propagation, *Escherichia coli* DH5α (Invitrogen, Shanghai, China) was cultured at 37 °C in Luria–Bertani broth plus kanamycin or ampicillin (100 µg mL^−1^). *Agrobacterium tumefaciens* AGL-1 used for fungal transformation was cultured at 28 °C in Luria–Bertani medium plus kanamycin (100 µg mL^−1^). *Myceliophthora thermophila* strain ATCC 42464 was purchased from the American Type Culture Collection, and *M. heterothallica* strain CBS 203.75 was obtained from the Centraalbureau voor Schimmelcultures. The *M. thermophila* and *M. heterothallica* strains were cultured on Vogel’s MM supplemented with 2% sucrose at 45 °C for 10 days to obtain conidia. For flask culture, 10-day-old conidia of *M. thermophila* strains were inoculated in 100 mL of liquid medium (with a final concentration of 1 × 10^6^ conidia mL^−1^) containing 1× Vogel’s salt with 2% (w/v) Avicel with or without 0.5% (w/v) peptone at 45 °C with shaking at 150 rpm.

### Construction of Cas9 and sgRNA expression plasmids

All primer sequences used in this study are listed in Additional file 7: Table S1. All PCR products were amplified using Phusion high-fidelity DNA polymerase (Thermo Fisher, Waltham, MA, USA). A codon-optimized *Cas9* gene with attached *hac*-*1* (MYCTH_2310995) nuclear localization signals (NLS-Cas9-NLS) was synthesized by Life Technologies (Invitrogen) for expression in *M. thermophila* and *M. heterothallica*. The synthetic NLS-Cas9-NLS, the strong constitutive *tef1* (MYCTH_2298136) promoter of *M. thermophila*, and the *TtprC* terminator were amplified using paired primers (Additional file 7: Table S1). With the aid of a NEB Gibson assembly kit, these amplification products were assembled to form a P*tef1*-*Cas9*-T*tprC* cassette (Additional file 8) and inserted into a p0380-bar plasmid [56] carrying the *bar* marker to generate the *Cas9*-expression vector p0380-*bar*-P*tef1*-*Cas9*-T*tprC*. A Gibson assembly kit was also used to construct a *Cas9*-eGFP-expression vector (p0380-*bar*-P*tef1*-*Cas9*-*eGFP*-T*tprC*) in which enhanced GFP (e*GFP*) was fused to *Cas9* as a reporter gene (Additional file 8). To generate sgRNA expression plasmids, an sgRNA scaffold was synthesized by Life Technologies. To express sgRNA in protoplasts, the *M. thermophila* U6 promoter was amplified from ATCC 42464 genomic DNA using the primer pair U6-F/R (Additional file 7: Table S1) and then fused to the sgRNA scaffold fragment by fusion PCR using U6-F and gRNA-R (Additional file 7: Table S1). The resulting fusion fragment was cloned into a pJET1.2/blunt cloning vector to create the corresponding plasmid U6p-sgRNA (Additional file 9).

To select for specific sgRNAs targeting *amdS* (GenBank number: M16371.1), *cre*-*1* (MYCTH_2310085), *res*-*1* (MYCTH_2302052), *gh1*-*1* (MYCTH_115968), and *alp*-*1* (MYCTH_2303011), all sgRNA target sites in the genome of *M. thermophila* were identified using the sgRNACas9 tool [57]. sgRNA target sites with high scores were chosen and the corresponding oligos were ordered (Additional file 7: Table S1). All protospacer sequences used to target the five different genes are presented in Table 1. A target-directed *M. thermophila* U6 promoter-driven sgRNA was created by overlapping PCR with the primers given in Additional file 7: Table S1 and cloned into a pJET1.2/blunt cloning vector, which yielded the corresponding plasmids U6p-*amdS*-sgRNA, U6p-*cre1*-sgRNA, U6p-*res1*-sgRNA, U6p-*gh1*-*1*-sgRNA, and U6p-*alp1*-sgRNA (Additional file 9).

### Donor DNA construction

For construction of gene deletion substrates, the P*trpC*-*neo* cassette was amplified from a p0380-neo plasmid [12]. The 5′ and 3′ flanking fragments of *cre*-*1*, *res*-*1*, *gh1*-*1*, and *alp1* were separately amplified from *M. thermophila* genomic DNA via PCR with paired primers (Additional file 7: Table S1). The amplified 5′, 3′ and P*trpC*-*neo* fragments were assembled and ligated into a pJET1.2/blunt cloning vector using a NEB Gibson assembly kit to generate four donor DNA sequences: donor-*cre1*, donor-*res1*, donor-*gh1*-*1,* and donor-*alp1* (Additional file 10).

### Expression of Cas9 in *Myceliophthora thermophila*

Two *Cas9* expression vectors, p0380-*bar*-P*tef1*-*Cas9*-T*tprC* and p0380-*bar*-P*tef1*-*Cas9*-*eGFP*-T*tprC*, were transformed into WT *M. thermophila* strain ATCC 42464 via *Agrobacterium*-mediated transformation [12]. Colonies grown for 4 days on MM at 35 °C were screened for *bar* gene resistance using 100 µg mL^−1^ phosphinothricin (Sigma-Aldrich, St. Louis, MO, USA), followed by sequential identification via PCR analysis. The positive transformants from each construct were named Cas9OE and Cas9-gfp. Cas9OE-positive transformants selected as replicates were subjected to three consecutive rounds of sub-culture; their phenotypes, including secreted protein production, mycelial dry weights and lignocellulosic enzyme activities, were examined in parallel with the WT strain.

### Subcellular localization of Cas9-gfp in *Myceliophthora thermophila*

To localize GFP fusion proteins by microscopy, Cas9-gfp-positive transformants were inoculated in liquid MM containing 2% sucrose as the carbon source and grown for 24 h at 45 °C. Before imaging, hyphae were harvested and incubated with 1 mg mL^−1^ 4′, 6-diamidino-2-phenylindole for 15 min. The microscopic observation was performed using an Olympus BX51 fluorescence microscopy system, with the ImageJ software used for image processing.

### Transformation of *Myceliophthora thermophila* and *M. heterothallica* protoplasts

Transformation of *M. thermophila* and *M. heterothallica* protoplasts was performed according to a previously described procedure [44]. For *amdS* mutagenesis, the promoter P*tef1* (MYCTH_2298136) and the full-length acetamidase-encoding gene *amdS* (GenBank number: M16371.1) were amplified from genomic DNA of *M. thermophila* and the plasmid p3SR2 using the paired primers Ptef1-F2/R2 and amdS-F/R, respectively. The *amdS* gene was fused with P*tef1* by overlapping PCR using the primer pair Ptef1-F2/amdS-R. The P*tef1*-*amdS* cassette was then ligated into the *Eco*RI and *Hin*dIII sites of a pCAMBIA-0380 plasmid [56], thereby generating the expression plasmid p0380-P*tef1*-*amdS*. This constructed plasmid was transformed into the WT strain via *Agrobacterium*-mediated transformation. Colonies grown for 5 days at 35 °C were selected in medium containing 10 mM acetamide as the sole nitrogen source. Positive transformants were identified by PCR with paired primers (Additional file 7: Table S1). From these transformants, the *amdS* expression strain M1 was chosen and used for further CRISPR/Cas9 manipulation. Briefly, 10 µg of the Cas9-expression PCR cassette *bar*-P*tef1*-*Cas9*-T*tprC* and the gRNA expression PCR product U6p-*amdS*-sgRNA at a molar concentration ratio of 1:1 was co-transformed into protoplasts of the recipient strain M1. After transformation, *amdS* mutants were inoculated onto MM agar plates supplemented with 2 mg mL^−1^ FAA and 100 µg mL^−1^ phosphinothricin. After 3 days incubation at 35 °C, FAA-resistant mutants were isolated and tested for growth on acetamide medium, followed by identification and sequencing via PCR with paired primers (Additional file 7: Table S1).

For single-gene editing, 10 µg of the Cas9-expression PCR cassette *bar*-P*tef1*-*Cas9*-T*tprC*, gRNA expression PCR cassette U6p-*cre1*-sgRNA, and donor-*cre1* was mixed at a molar concentration ratio of 1:1:1 and added to the fungal protoplasts. Control experiments were performed by adding 10 µg of donor-*cre1* alone, or only the Cas9 cassette and donor-*cre1*, or only U6p-*cre1*-sgRNA and donor-*cre1* to the fungal protoplasts. Transformants were screened for *bar* resistance with phosphinothricin (100 µg mL^−1^) and *neo* resistance with G418 (40 µg mL^−1^), followed by PCR identification with paired primers (Additional file 7: Table S1).

For multiple genomic edits, the generated *amdS* mutant M2 containing a Cas9 expression chassis was used as a host. Multiple genomic modification involving the *cre*-*1*, *res*-*1*, *gh1*-*1,* and *alp*-*1* loci was performed in M2 protoplasts through co-transformation of two, three, or four sets of sgRNA expression cassettes and donor DNA fragments at the same molar concentration. The putative transformants were selected on MM supplemented with 100 µg mL^−1^ phosphinothricin and 40 µg mL^−1^ G418, followed by sequential identification via PCR with paired primers (Additional file 7: Table S1).

### Determining copy numbers by RT-qPCR

To determine the copy numbers of the integrated P*trpC*-*neo* marker gene in the transformants, the fungal genomic DNAs were extracted as described previously [58] and used as templates for RT-qPCR. The RT-qPCR method was essentially the same as that described by Abad et al. [59]. The qPCR was performed with SYBR Green Real-time PCR Master Mix (TOYOBO, Osaka, Japan) in a CFX96 Real-Time PCR Detection System (Bio-Rad, Hercules, CA, USA). The primers used for the genes are listed in Additional file 7: Table S1. The *actin* gene (MYCTH_2314852) was used as an internal control. Each 20 μL reaction contained 1 μL of diluted DNA as the template, 10 μL of 2× SYBR Green Real-time PCR Master Mix, 1 μL of each primer (0.4 M), and 7 μL of H_2_O. An equal volume of water instead of DNA was used as a negative control. The qPCR was performed as follows: 95 °C for 30 s, 40 cycles of 95 °C for 30 s, 55 °C for 30 s, and 72 °C for 30 s. A melt curve analysis was performed at the end of each run from 55 to 95 °C with a ramp speed of 0.5 °C to ensure specific sequence amplification of all primers and only one melting temperature on the melting curve. To determine the amplification efficiencies between 90 and 110% of all reactions, the genomic DNA and plasmid DNA samples were diluted serially to construct standard curves and then subjected three times to RT-qPCR.

### Protein and enzyme activity measurements

Total extracellular protein contents in the culture supernatants were measured using a Bio-Rad DC protein assay kit (Bio-Rad) based on absorbance at 595 nm, with bovine serum albumin used as the standard. For protein gel electrophoresis, 30-µL aliquots of concentrated culture supernatants were subjected to sodium dodecylsulfate-polyacrylamide gel electrophoresis on Novex NuPAGE pre-cast protein gels (Thermo Fisher Scientific). Endoglucanase activity in the culture supernatants was determined using an azo-cm-cellulose assay kit (Megazyme, Wicklow, Ireland) according to the manufacturer’s protocol. Endo-1,4-β-xylanase activities were assayed with an azo-xylan kit (Megazyme) following the method specified by the manufacturer. FPA activities were assayed with Whatman No.1 filter paper as the substrate. The enzyme reactions were performed in 50 mM citrate buffer (pH 4.8) at 50 °C for 60 min, using the DNS method to quantify the released reducing sugar. Exoglucanase activity was assayed according to the method described by Zou et al. [60] and measured at 50 °C using 1.0 mg mL^−1^ *p*-nitrophenyl-β-D-cellobioside (Sigma-Aldrich) as the substrate in 50 mM citrate buffer (pH 4.8) containing 1 mg mL^−1^ d-glucono-1,5-σ-lactone. Each reaction mixture containing 250 µL of properly diluted enzyme and 250 µL of 1.0 mg mL^−1^ substrate in 50 mM citrate buffer (pH 4.8) was incubated for 10 min at 50 °C, and the reaction was terminated by adding 500 µL of 1 M Na_2_CO_3_. Released *p*-nitrophenol (*p*NP) was measured at an absorbance of 420 nm. Inactive enzyme, which was boiled at 100 °C for 10 min, was used as a control. *p*NP was used for the standard curve. In the exoglucanase activity analyses, one unit (U) of enzymatic activity was defined as the amount of 1 μmol glucose or *p*NP released by 1 mL of enzyme from the substrate per minute under the standard assay conditions. All estimates were performed in three repeated assays. The statistical significance of differences among WT and mutant strains was assessed by one-way analysis of variance.

## Additional files

**Additional file 1: Figure S1.** Phenotype of WT and constitutive Cas9-expressing strains. (A) Sodium dodecylsulfate-polyacrylamide gel electrophoresis of secreted protein of the WT and Cas9-OE after 4 days of culture on 2% Avicel. (B-E) Assays for protein concentration and xylanase, endoglucanase and exoglucanase activities of Cas9-OE and the WT in inducing medium with 2% Avicel after 3 and 4 days culture. (F) Mycelial dry weights of Cas9-OE and the WT from cultures on sucrose and Avicel. No significant difference (Tukey’s HSD, *p* > 0.5) was observed between Cas9-OE and the WT in the above assays.

**Additional file 2: Figure S2.** Verification of *cre*-*1* gene deletions in selected transformants with co-transformation of only Cas9 and donor DNA, only sgRNA and donor DNA, or donor DNA alone. (A) Schematic of homologous recombination (HR) of the target gene *cre*-*1*. (B-G) PCR analysis of *cre*-*1* deletion in *M. thermophila* (B-D) and *M. heterothallica* (E-G) with one primer (cre1-out-F) located upstream of the 5′ flanking region of the genomic DNA and the other (cre1-in-R) located in the 3′ flanking region of the genomic DNA. The expected length of disrupted transformants was 1.9 kb, while that of the host strain, used as a negative control, was 1.0 kb (rightmost lane).

**Additional file 3: Figure S3.** Verification of double-gene deletions of *cre*-*1* and *res*-*1*, *cre*-*1* and *res*-*1*, and *gh1*-*1* and *res*-*1* in selected transformants with co-transformation of multiple fragments. (A) Schematic of homologous recombination (HR) of target genes mediated by Cas9, sgRNAs and donor DNA. (B-D) PCR analysis of double-gene deletion of *cre*-*1* and *gh1*-*1* (B), *cre*-*1* and *res*-*1* (C) and *gh1*-*1* and *res*-*1* (D) in selected transformants using one primer (cre1/gh1-1/res1-out-F) located upstream of the 5′ flanking region of the genomic DNA and the other primer (cre1/gh1-1/res1-in-R) located in the 3′ flanking region of the genomic DNA. The expected length of disrupted transformants was 1.9 kb, while that of the host strain (rightmost lane) was 1.0 kb.

**Additional file 4: Figure S4.** Verification of triple-gene deletions of *cre*-*1*, *res*-*1*, *gh1*-*1* and *alp*-*1* in selected transformants. (A) Schematic of homologous recombination (HR) of *cre*-*1*, *res*-*1* and *gh1*-*1* mediated by Cas9, sgRNAs and donor DNA. (B) PCR analysis of triple-gene deletion of *cre*-*1*, *res*-*1* and *gh1*-*1* in selected transformants with specific paired primers (cre1/gh1-1/res1-out-F and cre1/gh1-1/res1-in-R). The expected length of disrupted transformants was 1.9 kb, while that of the host strain (rightmost lane) was 1.0 kb.

**Additional file 5: Figure S5.** Verification of quadruple-locus deletion of *cre*-*1*, *res*-*1*, *gh1*-*1* and *alp*-*1* in selected transformants. (A) Schematic of homologous recombination (HR) of target genes mediated by Cas9, sgRNA and donor DNA. (B) PCR analysis of quadruple-gene deletion of *cre*-*1*, *res*-*1*, *gh1*-*1* and *alp*-*1* in selected transformants with paired primers (cre1/res1/gh1-1/alp1-out-F and cre1/res1/gh1-1/alp1-in-R). The expected length of disrupted transformants was 1.9 kb, while that of the host strain M2 (rightmost lane) was 1.0 kb.

**Additional file 6: Figure S6.** Determination of P*trpC*-*neo* cassette copy numbers in the disrupted mutants by RT-qPCR analysis.

**Additional file 7: Table S1.** List of PCR primers used in this study.

**Additional file 8.** Nucleotide sequences of Cas9 expression cassettes.

**Additional file 9.** Nucleotide sequences of sgRNA expression cassettes.

**Additional file 10.** Nucleotide sequences of donor DNA fragments.

## Abbreviations

CRISPR: clustered regularly interspaced short palindromic repeats
HR: homologous recombination
sgRNA: single chimeric guide RNA
PAM: protospacer adjacent motif
DSB: double-strand break
NHEJ: nonhomologous end joining
HDR: homology-directed repair
eGFP: enhanced green fluorescence protein
FAA: fluoroacetamide
CCR: carbon catabolite repression
WT: wild type
FPA: filter paper activity
RT-qPCR: real-time quantitative PCR
