# Supplementary figures and images for: Development of a genome-editing CRISPR/Cas9 system in thermophilic fungal Myceliophthora species and its application to hyper-cellulase production strain engineering
Source: Biotechnol Biofuels. 2017 Jan 3;10:1. doi: 10.1186/s13068-016-0693-9 (PMC5209885; doi:10.1186/s13068-016-0693-9)

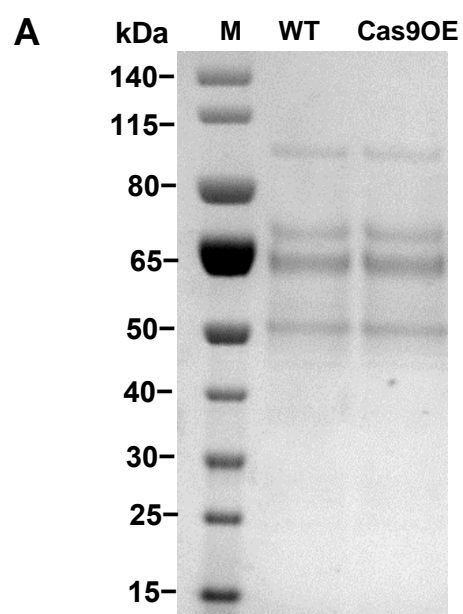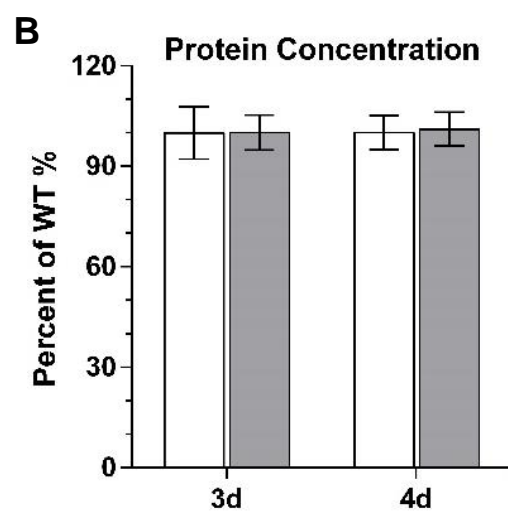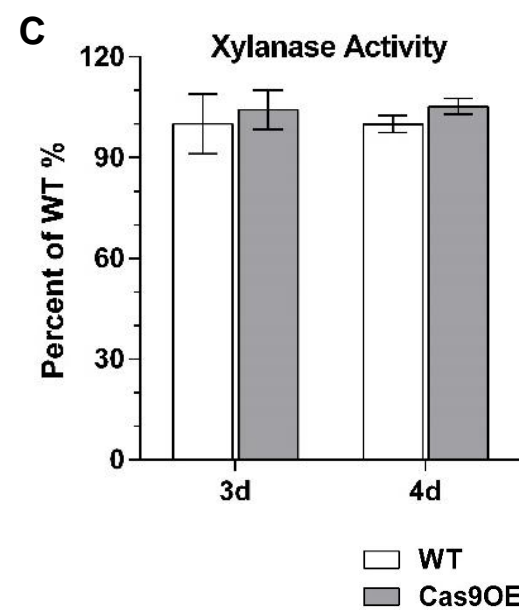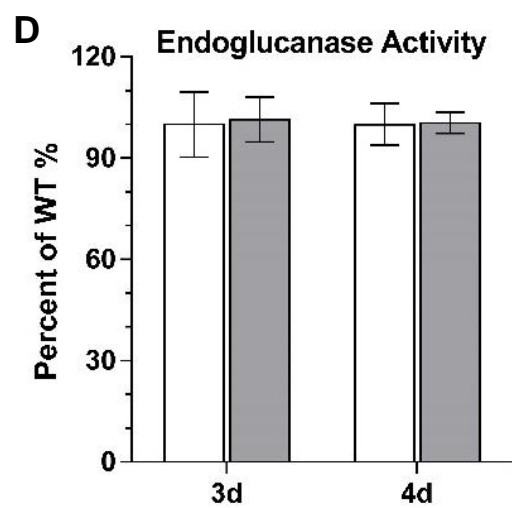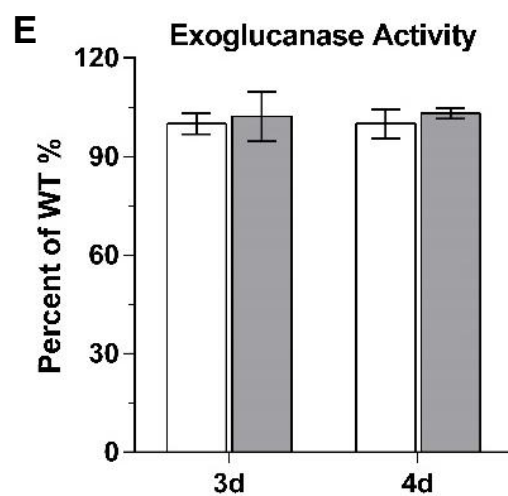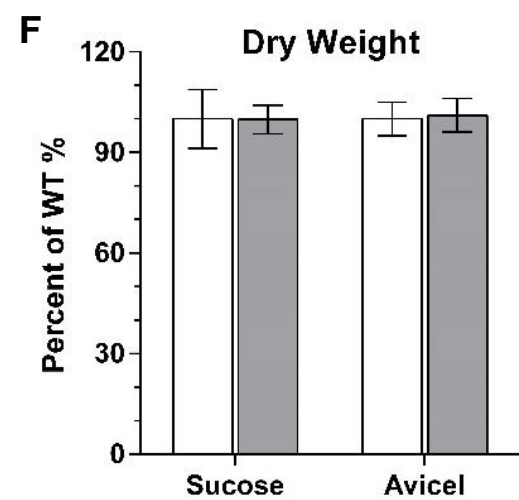

Supplement: Supplementary file 1 — Additional file 1: Figure S1. Phenotype of WT and constitutive Cas9-expressing strains. (A) Sodium dodecylsulfate-polyacrylamide gel electrophoresis of secreted protein of the WT and Cas9-OE after 4 days of culture on 2% Avicel. (B-E) Assays for protein concentration and xylanase, endoglucanase and exoglucanase activities of Cas9-OE and the WT in inducing medium with 2% Avicel after 3 and 4 days culture. (F) Mycelial dry weights of Cas9-OE and the WT from cultures on sucrose and Avicel. No significant difference (Tukey’s HSD, p > 0.5) was observed between Cas9-OE and the WT in the above assays. [file 13068_2016_693_MOESM1_ESM.pdf]

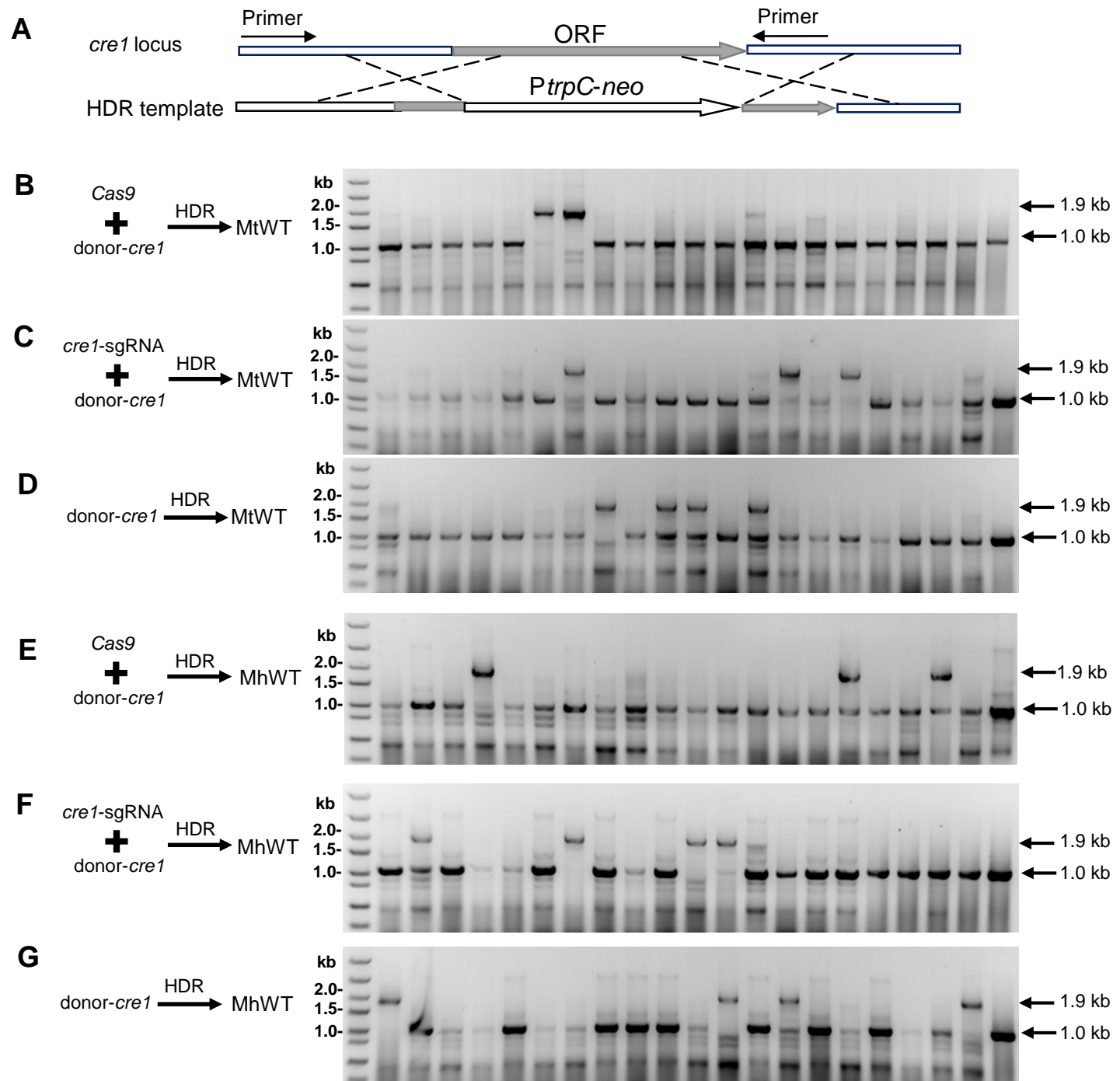

Supplement: Supplementary file 2 — Additional file 2: Figure S2. Verification of cre-1 gene deletions in selected transformants with co-transformation of only Cas9 and donor DNA, only sgRNA and donor DNA, or donor DNA alone. (A) Schematic of homologous recombination (HR) of the target gene cre-1. (B-G) PCR analysis of cre-1 deletion in M. thermophila (B-D) and M. heterothallica (E-G) with one primer (cre1-out-F) located upstream of the 5′ flanking region of the genomic DNA and the other (cre1-in-R) located in the 3′ flanking region of the genomic DNA. The expected length of disrupted transformants was 1.9 kb, while that of the host strain, used as a negative control, was 1.0 kb (rightmost lane). [file 13068_2016_693_MOESM2_ESM.pdf]

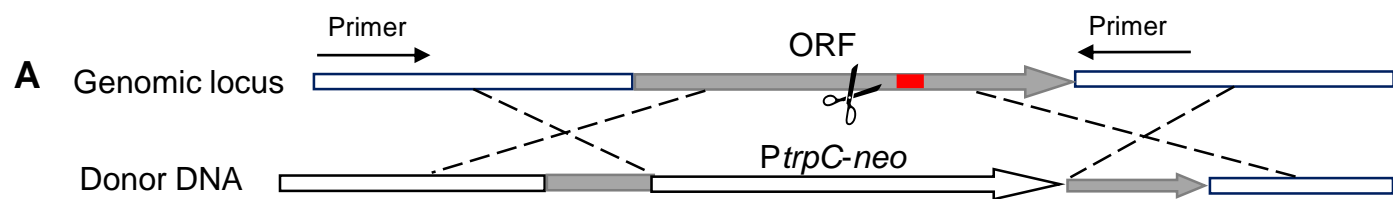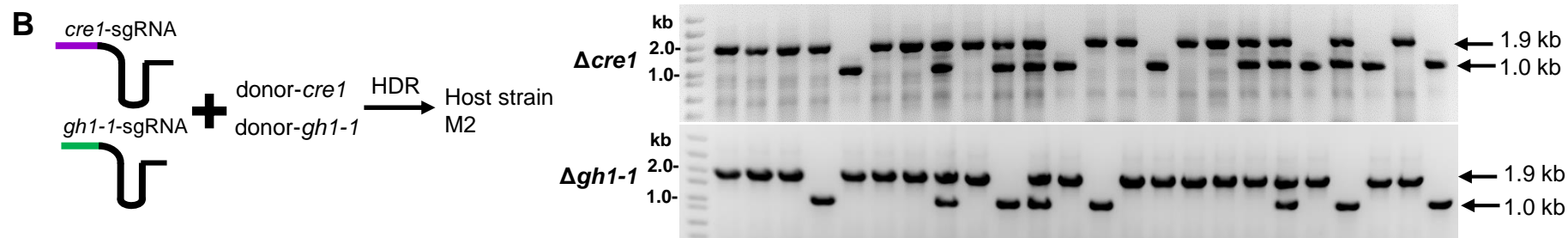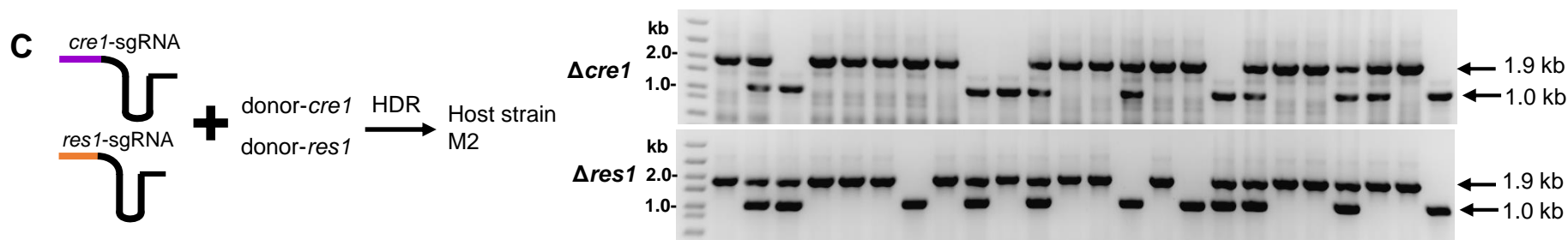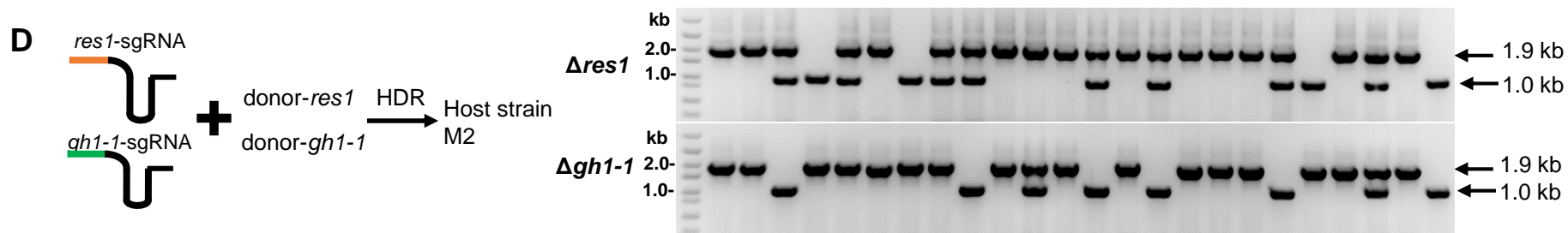

Supplement: Supplementary file 3 — Additional file 3: Figure S3. Verification of double-gene deletions of cre-1 and res-1, cre-1 and res-1, and gh1-1 and res-1 in selected transformants with co-transformation of multiple fragments. (A) Schematic of homologous recombination (HR) of target genes mediated by Cas9, sgRNAs and donor DNA. (B-D) PCR analysis of double-gene deletion of cre-1 and gh1-1 (B), cre-1 and res-1 (C) and gh1-1 and res-1 (D) in selected transformants using one primer (cre1/gh1-1/res1-out-F) located upstream of the 5′ flanking region of the genomic DNA and the other primer (cre1/gh1-1/res1-in-R) located in the 3′ flanking region of the genomic DNA. The expected length of disrupted transformants was 1.9 kb, while that of the host strain (rightmost lane) was 1.0 kb. [file 13068_2016_693_MOESM3_ESM.pdf]

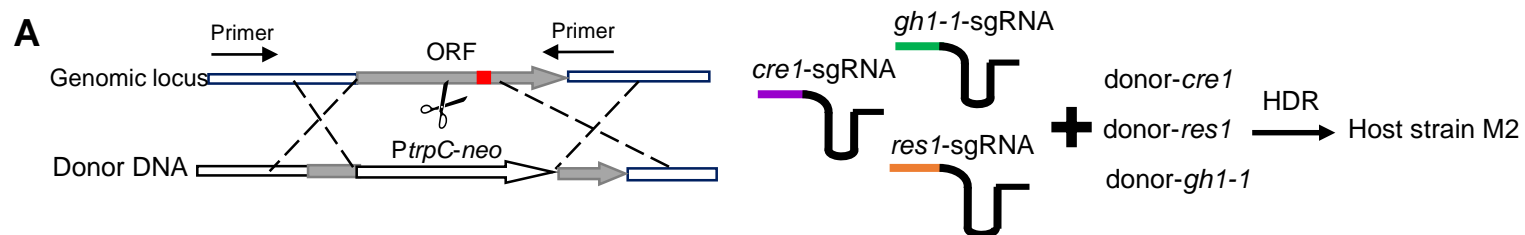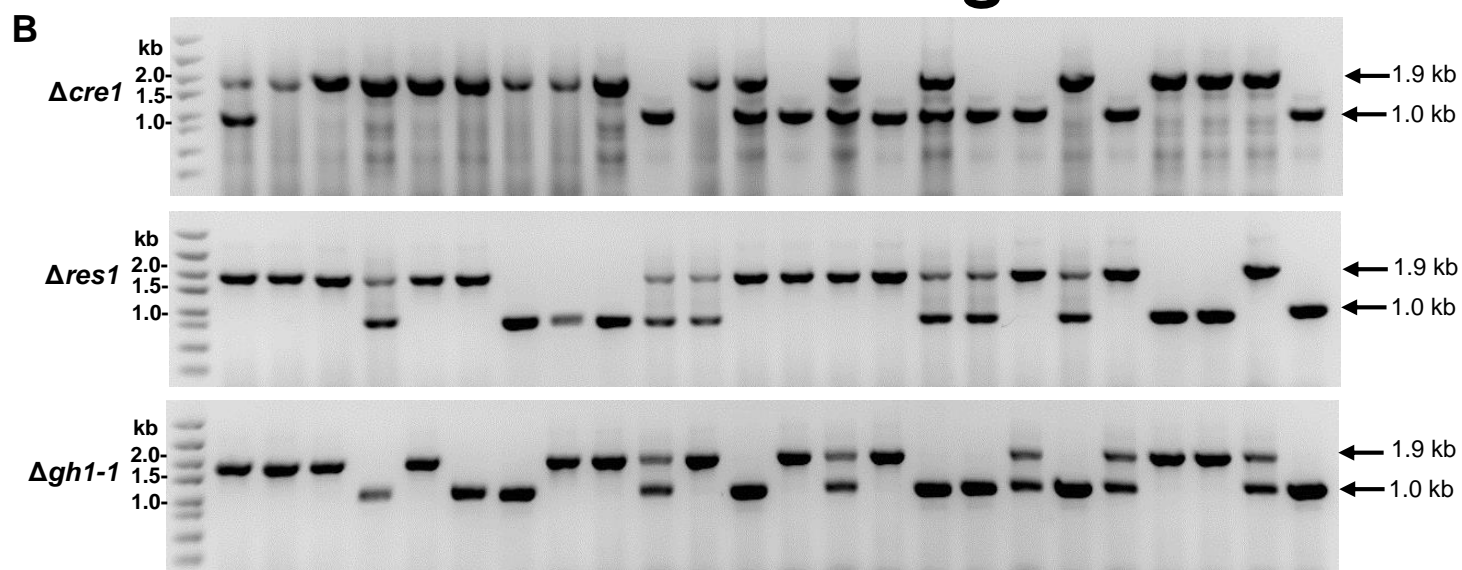

Supplement: Supplementary file 4 — Additional file 4: Figure S4. Verification of triple-gene deletions of cre-1, res-1, gh1-1 and alp-1 in selected transformants. (A) Schematic of homologous recombination (HR) of cre-1, res-1 and gh1-1 mediated by Cas9, sgRNAs and donor DNA. (B) PCR analysis of triple-gene deletion of cre-1, res-1 and gh1-1 in selected transformants with specific paired primers (cre1/gh1-1/res1-out-F and cre1/gh1-1/res1-in-R). The expected length of disrupted transformants was 1.9 kb, while that of the host strain (rightmost lane) was 1.0 kb. [file 13068_2016_693_MOESM4_ESM.pdf]

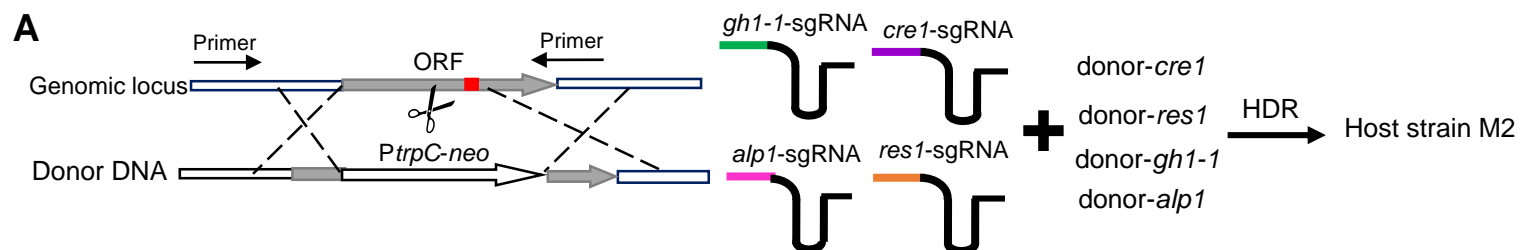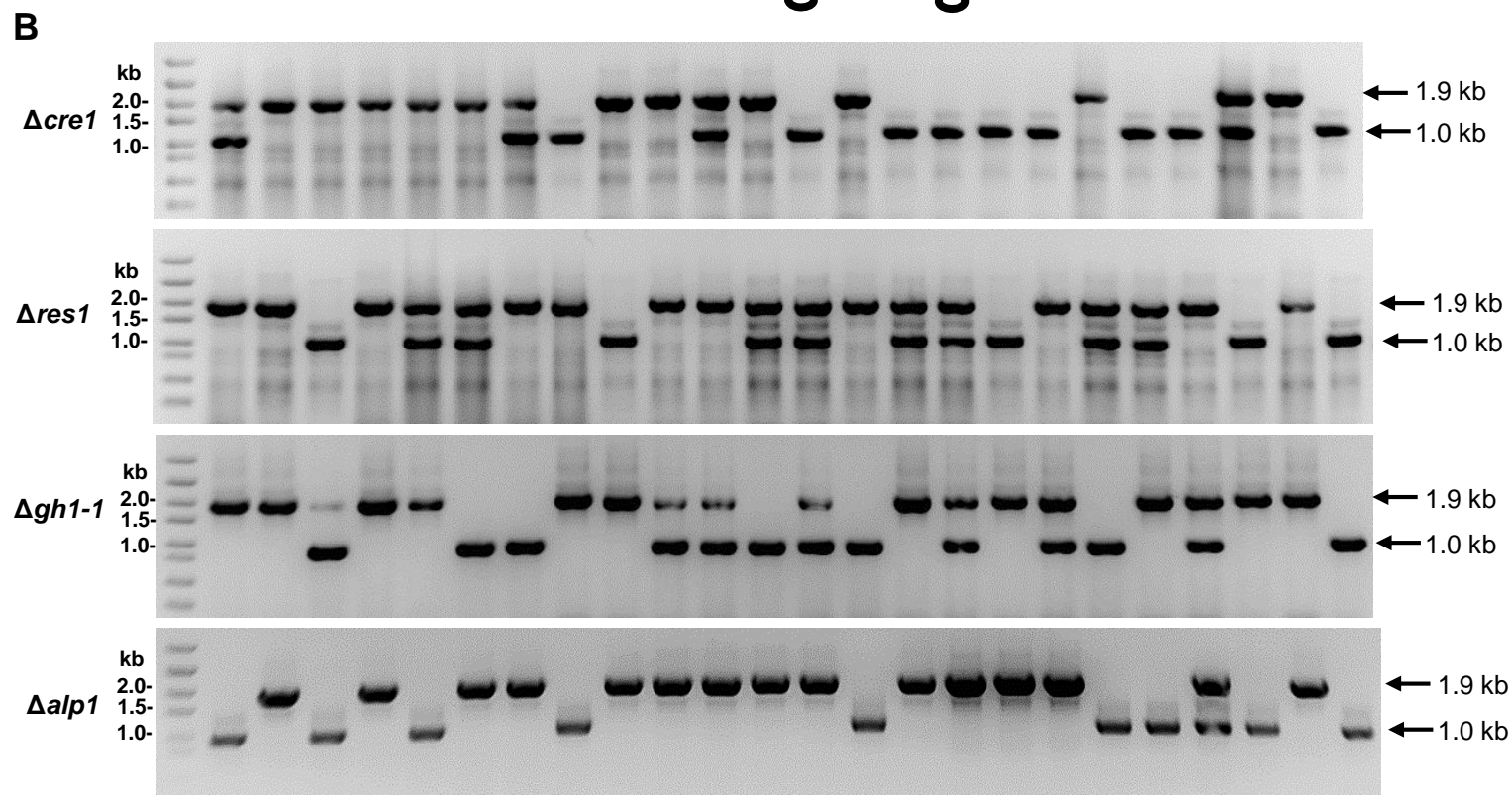

Supplement: Supplementary file 5 — Additional file 5: Figure S5. Verification of quadruple-locus deletion of cre-1, res-1, gh1-1 and alp-1 in selected transformants. (A) Schematic of homologous recombination (HR) of target genes mediated by Cas9, sgRNA and donor DNA. (B) PCR analysis of quadruple-gene deletion of cre-1, res-1, gh1-1 and alp-1 in selected transformants with paired primers (cre1/res1/gh1-1/alp1-out-F and cre1/res1/gh1-1/alp1-in-R). The expected length of disrupted transformants was 1.9 kb, while that of the host strain M2 (rightmost lane) was 1.0 kb. [file 13068_2016_693_MOESM5_ESM.pdf]

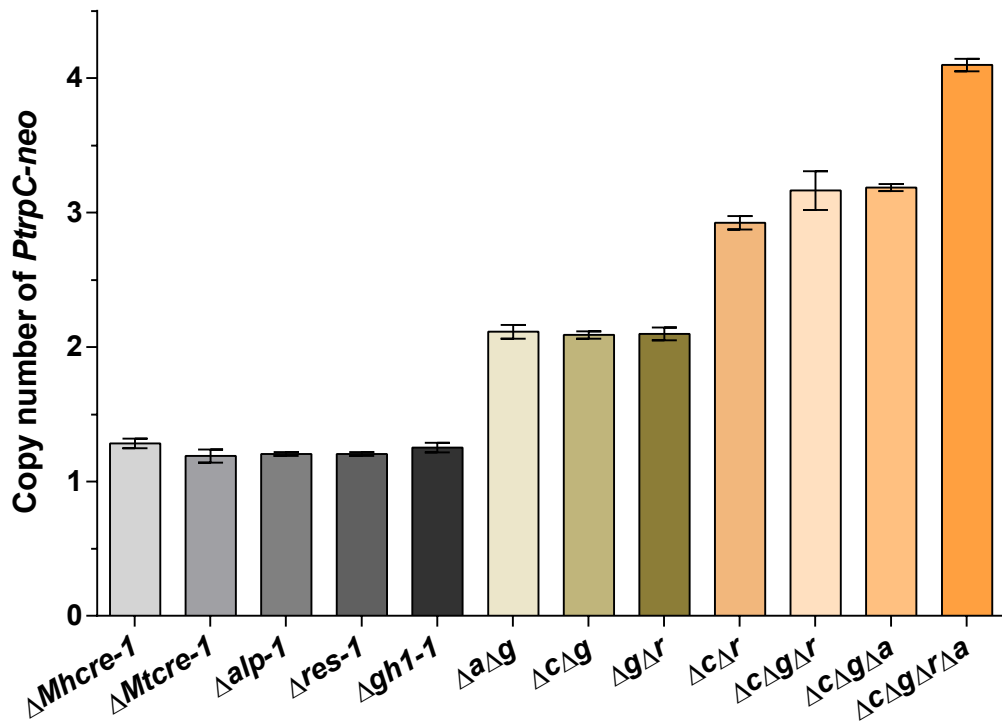

Supplement: Supplementary file 6 — Additional file 6: Figure S6. Determination of PtrpC-neo cassette copy numbers in the disrupted mutants by RT-qPCR analysis. [file 13068_2016_693_MOESM6_ESM.pdf]
