# Supplementary material for: Development of a genome-editing CRISPR/Cas9 system in thermophilic fungal Myceliophthora species and its application to hyper-cellulase production strain engineering
Source: Biotechnol Biofuels. 2017 Jan 3;10:1. doi: 10.1186/s13068-016-0693-9 (PMC5209885; doi:10.1186/s13068-016-0693-9)
Supplement: Supplementary file 7 — Additional file 7: Table S1. List of PCR primers used in this study. [file 13068_2016_693_MOESM7_ESM.pdf]

Additional file 1: Supplementary Table S1. List of PCR primers used in this study.

Construction of Cas9 expressing vectors		
Cloning of Cas9	Cas9-F	GCCAGTTTCGTTCTTCAGAAAGCTTATGGACTACAAGGACCATGATGGCG
Cloning of Cas9	Cas9-R	TGATGATTTCAGTAACGTTAAGTGGATCCTCATCACTCATCTTCTGTTTTGGCAC
Cloning of *tef1* promoter	Ptef1-F1	CTAGGCCACCATGTTGGGCCCGGCGCGCCGAATTCTCCTCCGAGGTTCGACATCA
Cloning of *tef1* promoter	Ptef1-R1	CGCCATCATGGTCCTTGTAGTCCATAAGCTTTCTGAAGAACGAAACTGGC
Cloning of *TtprC* terminator	TtprC-F	GTGCCAAAACAGAAGATGAGTGATGAGGATCCACTTAACGTTACTGAAATCATCA
Cloning of *TtprC* terminator	TtprC-R	TCCTGCCCGTCACAGAGATTTGACCATGGGAGCTCCCTCTAAACAAGTGTACCTG
Cloning of GFP	GFP-F	TCTCTCCCAGCTCGGCGGCGACGAATTCATGGTGAGCAAGGGCGAGGAGCTGTTC
Cloning of GFP	GFP-R	TGATGATTTCAGTAACGTTAAGTGGATCCTTACTCATCTTCTGTTTTGGCACGTTTCCTC GGAGGCTTGTACAGCTCGTCCATGC
Construction of sgRNA expressing plasmids		
Cloning of U6 promoter	U6p-F	AGGATCGGTGGAGTGAAGTTCGGAA
Cloning of U6 promoter	U6p-amdS-R	CTAAAACGACCCTCCATGCTGTTCGCCGAGGAAAGAAAGAAAAGAAGAG
Cloning of U6 promoter	U6p-cre1-R	CTAAAACCTGCAGACTTTGCGCGTTGCGAGGAAAGAAAGAAAAGAAGAG
Cloning of U6 promoter	U6p-res1-R	CTAAAACGGTACGAGGGCTCATAGGGCGAGGAAAGAAAGAAAAGAAGAG
Cloning of U6 promoter	U6p-gh1-1-R	CTAAAACGGGATGGCGCAGAATGTGTCGAGGAAAGAAAGAAAAGAAGAG
Cloning of U6 promoter	U6p-alp1-R	CTAAAACCTGAACTTGCCGCGGTAGACGAGGAAAGAAAGAAAAGAAGAG
amdS-gRNA 5′	g-amdS-F	TCCTCGGCGAACAGCATGGAGGGTCGTTTTAGAGCTAGAAATAGCAAGTT
cre1-gRNA 5′	g-cre1-R	TCCTCGCAACGCGCAAAGTCTGCAGGTTTTAGAGCTAGAAATAGCAAGTT
res1-gRNA 5′	g-res1-R	TCCTCGCCCTATGAGCCCTCGTACCGTTTTAGAGCTAGAAATAGCAAGTT
gh1-1-gRNA 5′	g-gh1-1-R	TCCTCGACACATTCTGCGCCATCCCGTTTTAGAGCTAGAAATAGCAAGTT
alp-1-gRNA 5′	g-alp1-R	TCCTCGTCTACCGCGGCAAGTTCAGGTTTTAGAGCTAGAAATAGCAAGTT
gRNA 3′	gRNA-R	AAAAAAAGCACCGACTCGGTGCCACTT
Construction of *amdS*-expression strains		
Cloning of *tef* promoter	Ptef1-F2	CCGGAATTCCAAAAAAAAAGAGAGATGAGTGGGC
Cloning of *tef* promoter	Ptef1-R2	CCAGTTCTTCCCAGGATTGAGGCATTCTGAAGAACGAAACTGGCGACTTG
Cloning of *amdS*	amdS-F	CAAGTCGCCAGTTTCGTTCTTCAGAATGCCTCAATCCTGGGAAGAACTGG
Cloning of *amdS*	amdS-R	CCCAAGCTTGCCATCCAGAGTCACCTGTGGCAGC
Donor DNA construction		
cre1-upstream	cre1-5′-F	TTGGGCCCGGCGCGCCGAATTCCCGGGGATCCACCGGGGTAAGGTACTCTGTAAGTT
cre1-upstream	cre1-5′-R	GCTCCTTCAATATCAGTTAACGTCGTATGTCCACCCTCCCAAGCGGAGTT
Cloning of PtrpC-neo	neo-cre1-F	AACTCCGCTTGGGAGGGTGGACATACGACGTTAACTGATATTGAAGGAGC
Cloning of PtrpC-neo	neo-cre1-R	AAGCGTTTGCTACACCCGGGGAACTTCAGAAGAACTCGTCAAGAAGGCGA
cre1-downstream	cre1-3′-F	TCGCCTTCTTGACGAGTTCTTCTGAAGTTCCCCGGGTGTAGCAAACGCTT
cre1-downstream	cre1-3′-R	CAGATCTACCATGGTGGACTCCTCTTAAAGCTTTGGGGTGTGGTCCGGCGTCGGGGAC
res1-upstream	res1-5′-F	TTGGGCCCGGCGCGCCGAATTCCCGGGGATCCAACCAACCCTCCTTCGACTTTCCGG
res1-upstream	res1-5′-R	GCTCCTTCAATATCAGTTAACGTCGACGTCCGGGTGAATTAAGGAGGGAT
Cloning of PtrpC-neo	neo-res1-F	ATCCCTCCTTAATTCACCCGGACGTCGACGTTAACTGATATTGAAGGAGC
Cloning of PtrpC-neo	neo-res1-R	AAGTGGGACTGGTACGAGACAAGACTCAGAAGAACTCGTCAAGAAGGCGA
res1-downstream	res1-3′-F	TCGCCTTCTTGACGAGTTCTTCTGAGTCTTGTCTCGTACCAGTCCCACTT
res1-downstream	res1-3′-R	CAGATCTACCATGGTGGACTCCTCTTAAAGCTTGGTGCTCGTAAAAGTCTTGTGCGTT
gh1-1-upstream	gh1-1-5′-F	TTGGGCCCGGCGCGCCGAATTCCCGGGGATCCACTATCAACAGCCCCCCGGATTGCT

gh1-1-upstream	gh1-1-5′-R	GCTCCTTCAATATCAGTTAACGTCGTGGAGGGGCCACGGCCATCCTCGTT
Cloning of PtrpC-neo	neo gh1-1-F	AACGAGGATGGCCGTGGCCCCTCCACGACGTTAACTGATATTGAAGGAGC
Cloning of PtrpC-neo	neo- gh1-1-R	TTGCACTTGGGGATGGCCTTGAACATCAGAAGAACTCGTCAAGAAGGCGA
gh1-1-downstream	gh1-1-3′-F	TCGCCTTCTTGACGAGTTCTTCTGATGTTCAAGGCCATCCCCAAGTGCAA
gh1-1-downstream	gh1-1-3′-R	CAGATCTACCATGGTGGACTCCTCTTAAAGCTTGCAGCCAGAAGGACTGCGTCTCGGG
alp1-upstream	alp1-5′-F	TTGGGCCCGGCGCGCCGAATTCCCGGGGATCCCCTTCCTAGTCCTCGCAGCAAACTC
alp1-upstream	alp1-5′-R	CAAGCTCCGCTCCAAGGCCGACCACCGACGTTAACTGATATTGAAGGAGC
Cloning of PtrpC-neo	neo-alp1-F	CAAGCTCCGCTCCAAGGCCGACCACCGACGTTAACTGATATTGAAGGAGC
Cloning of PtrpC-neo	neo-alp1-R	TGTCGTAGGTGTAGGTGGTGGAGCCTCAGAAGAACTCGTCAAGAAGGCGA
alp1-downstream	alp1-3′-F	TCGCCTTCTTGACGAGTTCTTCTGAGGCTCCACCACCTACACCTACGACA
alp1-downstream	alp1-3′-R	CAGATCTACCATGGTGGACTCCTCTTAAAGCTTGAGTTGGCGGCGTTCTGGTTCTCGT
PCR analysis of point mutations and gene deletion		
Sequencing and verifying *amdS*	amdS-in-F	CTCCCCATCTCTCTCAAAGACCAGC
Sequencing and verifying *amdS*	amdS-in-R	GCTCACTAACCGCCTTGAAACTCTC
PCR detecting *cre1* disruption	cre1-out-F	ATACAGTACCTCTGCACAACCATCC
PCR detecting *cre1* disruption	cre1-in-R	AGTTGGGATTGTTGTGTATCCTCGA
PCR detecting *res1* disruption	res1-out-F	AACCCCAGGCATCGTAGATCAGGGC
PCR detecting *res1* disruption	res1-in-R	GCATATGAGCCTTGAGGTCCTTGAA
PCR detecting *gh1-1* disruption	gh1-1-out-F	CCTCAGTCTCCGCAATATCGTGCAT
PCR detecting *gh1-1* disruption	gh1-1-in-R	GAAGTAGCCCGTGTTGTAGCCCAGG
PCR detecting *alp1* disruption	alp1-out-F	TTCTGGCCTGCCCTTTTCTTTCAAC
PCR detecting *alp1* disruption	alp1-in-R	GCCCCTTCTTCCGAAAGGGGAGGTA
Determining P*trpC-neo* copy numbers by RT-qPCR		
RT-qPCR of *actin*	actin-RT-F	AACGCTCCTGCCTTCTAC
RT-qPCR of *actin*	actin-RT-R	GTAACACCATCACCAGAGTC
RT-qPCR of PtrpC-neo	PtrpC-RT-F	CATACCTTCTTAAGTTCG
RT-qPCR of PtrpC-neo	neo-RT-R	GCATCAGAGCAGCCGATT
