# Supplementary material for: Development of a genome-editing CRISPR/Cas9 system in thermophilic fungal Myceliophthora species and its application to hyper-cellulase production strain engineering
Source: Biotechnol Biofuels. 2017 Jan 3;10:1. doi: 10.1186/s13068-016-0693-9 (PMC5209885; doi:10.1186/s13068-016-0693-9)
Supplement: Supplementary file 8 — Additional file 8. Nucleotide sequences of Cas9 expression cassettes. [file 13068_2016_693_MOESM8_ESM.pdf]

Additional file 2: Nucleotide sequence of the Cas9 expression cassette. Purple letters indicate the *tef* promoter. Blue letters indicate the 3 × FLAG tag. Orange letters indicate the nuclear localization signal. Red letters indicate the *Cas9* gene. Green letters indicate the *GFP* gene. Gray letters indicate the TtrpC terminator.

> Cas9 expression cassette

TCCTCCGAGGTTCGACATCAGGGTTCGTCATAGGGAGTGAAACACCCGCCATGATTCCGTAGCCGCG
CGCGAAGATACGAAGCAGATATTTCACGGACATGGCGGAGATACTTGTTTCCCGTACTAAGGTAGTC
ATGTCGGAGACATCTGAACGACAGAGCTGGCCAAGAGAACCGACCAGTTGCCCCAGGACGATCTA
GACAAAAAAAAAGAGAGATGAGTGGGCCACTTTTGCCACAACATCGACGGCCCTGCGACCGCCCC
CAGGCAAACAAACAAACCGCCGAACAATAATACTTTTGTCATTTTAGGAGGAGCGTTGTATGGATAA
AAACAACATCTCGTTGCTGCAGAATGTGGACTTCAAACTTGCAGAAAATGGGAGGCGGATTTGCAT
GATCGGAGGGTAGTTGACTCACGCCGCAGGCTGCAAATCCGTCCTCCATTATTCCATGAACAACTTC
GTAAGGTTGGGCTGAGCGCCAATGCCTAACGGACCGGGGGCCACAGCGCAACGTCCCACTTAAAG
GCCAGCGTGACATGCCAGTTCCATACCAAGTAGTGGCACCAGAGGCGGCCAATGCTCAGTAAGGGC
AGGGAGGGAGGCTCAAACGATTGGCAAAAAGAGGGGCTTGCCAGTTCAGTTCCCTGTGCGAGCGC
GAGAGGGGCAGTTTCAAATCTGGAGGGGTGTGTTGCGCTGGTCTGAAGAGAAAGAGAAGACTGTA
CTTAATAATTGTTCAAAGAGTCCATCATCGCGTTGCGGACTCCTCTAGCTGTATTTAGAGCCCTATCAT
TACTTGTCGGGTGCGAATCAAAATACCGGGATGCAGCCCTCTGGCGATTTGCATGCGGTTGTGGAGG
AAGTGAAGCCTGAATCGCGGGGCTGGGCGGCAAAGCACGACGTGAAATTCCTGGCGAAATTCGAG
GGCTTGCCCCACCGTGGTTGAAGTTTTTGTGCTGCGTAACCCCACCAACCCGCCTTGCCCCTCCCGC
CTGCCCATAAAAACTTCGACCCCTCCTCAAATCTTCTTCGATTCTTCCTCTTCACTTCCTTCGTCGGC
ATACCTGATTCAAGCAATCACCTGCCACTTTCAAGTGCGTATACCATCATCGATACACTGGTTCTTGA
CAAGTACATCGTCTCTAACTTTCCTTTTTGCAGTTTTCATTAAGCGCAAGTCGCCAGTTTCGTTCTTC
AGAATGGACTACAAGGACCATGATGGCGATTACAAGGACCACGACATCGATTATAAGGATGATGA
TGACAAGCCTCCGAGGAAACGTGCCAAAACAGAAGATGAGATGGATAAGAAGTACTCCATCGGCC
TCGACATCGGCACCAACTCCGTCGGCTGGGCCGTCATCACCGATGAGTACAAGGTCCCTTCCAAGA
AGTTCAAGGTCCTCGGCAACACCGATCGCCATTCCATCAAGAAGAACCTGATCGGCGCCCTCCTGT
TCGATTCCGGCGAAACCGCCGAGGCCACCCGCCTTAAACGCACCGCCCGTCGCCGCTACACCCGCC
GCAAGAACCGCATCTGCTACCTCCAAGAAATCTTCTCCAACGAGATGGCCAAGGTCGATGATAGC
TTCTTCCACCGCCTCGAAGAGTCCTTCCTGGTCGAAGAGGATAAGAAGCACGAGCGCCATCCTATC
TTCGGCAACATCGTCGATGAGGTCGCCTACCATGAGAAGTACCCTACCATCTACCATCTCCGCAAG
AAGCTCGTCGATTCCACCGATAAGGCCGATCTCCGCCTCATCTACCTCGCCCTCGCCCATATGATC
AAGTTCCGCGGCCATTTCCTCATCGAGGGCGATCTCAACCCTGATAACTCCGATGTCGATAAGCTG
TTCATCCAGCTCGTCCAGACCTACAACCAGCTGTTCGAGGAAAACCCTATCAACGCCTCCGGCGTC
GATGCCAAGGCCATCCTCTCCGCTCGCCTCTCCAAGTCTCGCCGCCTTGAGAACCTTATCGCCCAG
CTCCCTGGCGAGAAGAAGAACGGCCTCTTCGGCAACCTGATCGCCCTCTCCCTCGGCCTCACCCCT
AACTTCAAGTCCAACTTCGATCTCGCCGAGGATGCCAAGCTCCAGCTCTCCAAGGATACCTACGAT
GATGATCTCGATAACCTCCTCGCCCAGATCGGCGATCAGTACGCCGATCTGTTCCTCGCCGCCAAG
AACCTCTCCGATGCCATCCTCCTCTCCGACATCCTCCGCGTCAACACCGAGATCACCAAGGCCCCT
CTGTCCGCCTCCATGATCAAGCGCTACGATGAGCATCATCAGGACCTCACCCTGCTCAAGGCCCTC
GTCCGCCAGCAGCTCCCTGAGAAGTACAAAGAGATTTTCTTCGATCAGTCCAAGAACGGCTACGCC
GGCTACATCGATGGCGGCGCTTCCCAAGAAGAGTTCTACAAGTTCATCAAGCCTATCCTTGAGAAG
ATGGATGGCACCGAGGAACTCCTCGTCAAGCTCAACCGCGAGGACCTCCTCCGCAAGCAGCGCAC

CTTCGATAACGGCTCCATCCCTCATCAAATCCATCTCGGCGAGCTGCATGCCATCTTGCGCCGCCA
AGAGGATTTCTACCCATTCCTCAAGGATAACCGCGAGAAGATCGAAAAGATTCTCACCTTCCGCAT
CCCTTACTACGTCGGCCCTCTCGCTCGCGGCAACTCCCGCTTCGCCTGGATGACCCGCAAGTCCGA
GGAAACCATCACCCCTTGGAACTTCGAGGAAGTCGTCGATAAGGGCGCCTCCGCCCAGTCCTTCAT
CGAGCGCATGACCAACTTCGATAAGAACCTCCCTAACGAGAAGGTCCTCCCTAAGCACTCCCTGCT
CTACGAGTACTTCACCGTCTACAACGAGCTGACCAAGGTCAAGTACGTCACCGAGGGTATGCGCA
AGCCTGCCTTCCTGTCCGGCGAGCAGAAGAAGGCCATCGTCGATCTGCTGTTCAAGACCAACCGCA
AGGTCACCGTCAAGCAGCTCAAAGAGGATTACTTCAAGAAAATCGAGTGCTTCGATTCCGTCGAG
ATCAGCGGCGTCGAGGACCGCTTCAACGCCTCCCTCGGAACCTACCATGATCTCCTCAAGATTATC
AAGGATAAGGATTTCCTCGACAACGAGGAAAACGAGGACATCCTTGAGGACATCGTCCTCACCCT
CACCCTCTTCGAGGACCGCGAAATGATCGAGGAACGCCTCAAGACCTACGCCCATCTCTTCGATGA
TAAGGTCATGAAGCAGCTCAAGCGCCGTCGCTACACCGGCTGGGGTCGCCTCTCCCGCAAGCTCAT
CAACGGCATCCGCGATAAGCAGTCCGGCAAGACTATCCTCGATTTCCTCAAGTCCGATGGCTTCGC
CAACCGCAACTTCATGCAGCTCATCCATGATGATTCCCTCACCTTCAAAGAGGACATCCAGAAGGC
CCAGGTCAGCGGCCAGGGCGATTCCCTCCATGAGCATATCGCCAACCTCGCCGGCTCCCCTGCCAT
CAAGAAGGGCATCCTCCAGACCGTCAAGGTCGTCGATGAGCTGGTCAAGGTCATGGGCCGCCATA
AGCCTGAGAACATCGTCATCGAGATGGCCCGCGAGAACCAGACCACCCAGAAGGGCCAGAAGAA
CTCCCGCGAGCGCATGAAGCGCATCGAGGAAGGCATCAAAGAGCTGGGCAGCCAAATCCTCAAAG
AGCATCCTGTCGAGAACACCCAGCTCCAGAACGAGAAGCTCTACCTCTACTACCTCCAGAACGGC
CGCGATATGTACGTCGATCAAGAGCTGGACATCAACCGCCTCTCCGATTACGATGTCGATCATATC
GTCCCTCAGTCCTTCCTGAAGGATGATTCCATCGATAACAAGGTCCTCACCCGCTCCGATAAGAAC
CGCGGCAAGTCCGATAACGTCCCTTCCGAAGAGGTCGTCAAGAAGATGAAGAACTACTGGCGCCA
GCTCCTCAACGCCAAGCTCATCACCCAGCGCAAGTTCGATAACCTCACCAAGGCCGAGCGCGGTG
GCCTCTCCGAGCTGGATAAGGCCGGCTTCATCAAGCGCCAGCTCGTCGAAACCCGCCAGATCACC
AAGCACGTCGCCCAAATCCTCGATTCCCGCATGAACACCAAGTACGATGAGAACGATAAGCTCAT
CCGCGAAGTCAAGGTCATCACCCTCAAGTCCAAGCTCGTCAGCGATTTCCGCAAGGATTTCCAGTT
CTACAAGGTCCGCGAGATCAACAACTACCATCATGCCCATGATGCCTACCTCAACGCCGTCGTCGG
CACCGCCCTCATCAAGAAGTACCCCAAGCTCGAATCCGAGTTCGTCTACGGTGATTACAAGGTCTA
CGATGTCCGCAAGATGATCGCCAAGTCCGAGCAAGAGATCGGCAAGGCTACCGCCAAGTACTTCT
TCTACTCCAACATCATGAATTTCTTCAAGACCGAAATCACCCTCGCCAACGGCGAAATCCGCAAGC
GCCCTCTCATCGAGACTAACGGCGAGACTGGCGAGATCGTCTGGGATAAGGGCCGCGATTTCGCC
ACCGTCCGCAAGGTCCTCTCCATGCCTCAGGTCAACATCGTCAAGAAAACCGAGGTCCAGACCGG
CGGCTTCTCCAAAGAGTCCATCCTCCCCAAGCGCAACTCCGATAAGCTGATCGCCCGCAAGAAGG
ATTGGGACCCTAAGAAGTACGGCGGCTTCGATTCCCCTACCGTCGCCTACTCCGTCCTCGTCGTCG
CCAAGGTCGAGAAGGGCAAGTCCAAGAAGCTCAAGTCCGTCAAAGAGCTGCTCGGCATCACTATT
ATGGAACGCTCCAGCTTCGAGAAGAACCCTATCGATTTCCTTGAGGCCAAGGGCTACAAAGAGGT
CAAGAAGGACCTCATCATCAAGCTCCCCAAGTACTCCCTGTTCGAGCTTGAGAACGGCCGCAAGC
GCATGCTCGCCTCCGCCGGTGAGCTTCAGAAGGGCAACGAGCTGGCCCTGCCTTCCAAGTACGTCA
ACTTCCTCTACCTCGCCTCCCATTACGAGAAGCTCAAGGGCTCCCCTGAGGATAACGAGCAGAAGC
AGCTGTTCGTCGAGCAGCATAAGCACTACCTCGATGAGATCATCGAGCAGATCAGCGAGTTCTCCA
AGCGCGTCATCCTCGCCGATGCCAACCTCGATAAGGTCCTGTCCGCCTACAACAAGCACCGCGATA
AGCCTATCCGCGAGCAGGCCGAGAACATCATCCATCTCTTCACCCTCACCAACCTCGGTGCCCCTG
CCGCCTTCAAGTACTTCGATACCACCATCGATCGCAAGCGCTACACCTCCACCAAAGAGGTCCTGG
ACGCCACCCTCATCCATCAGTCCATCACCGGCCTCTACGAAACCCGCATCGATCTCTCCCAGCTCG

GCGGCGACCCTCCGAGGAAACGTGCCAAAACAGAAGATGAGTGATGAGGATCCACTTAACGTTAC
TGAAATCATCAAACAGCTTGACGAATCTGGATATAAGATCGTTGGTGTCGATGTCAGCTCCGGAGTT
GAGACAAATGGTGTTCAGGATCTCGATAAGATACGTTCATTTGTCCAAGCAGCAAAGAGTGCCTTCT
AGTGATTTAATAGCTCCATGTCAACAAGAATAAAACGCGTTTTCGGGTTTACCTCTTCCAGATACAGC
TCATCTGCAATGCATTAATGCATTGACTGCAACCTAGTAACGCCTTACAGGCTCCGGCGAAGAGAAG
AATAGCTTAGCAGAGCTATTTTCATTTTCGGGAGACGAGATCAAGCAGATCAACGGTCGTCAAGAG
ACCTACGAGACTGAGGAATCCGCTCTTGGCTCCACGCGACTATATATTTGTCTCTAATTGTACTTTGA
CATGCTCCTCTTCTTTACTCTGATAGCTTGACTATGAAAATTCCGTCACCAGCNCCTGGGTTCGCAAA
GATAATTGCATGTTTCTTCCTTGAACTCTCAAGCCTACAGGACACACATTCATCGTAGGTATAAACCT
CGAAATCANTTCCTACTAAGATGGTATACAATAGTAACCATGCATGGTTGCCTAGTGAATGCTCCGTA
ACACCCAATACGCCGGCCGAAACTTTTTTACAACTCTCCTATGAGTCGTTTACCCAGAATGCACAGG
TACACTTGTTTAGAGG

> Cas9-GFP expression cassette
TCCTCCGAGGTTCGACATCAGGGTTCGTCATAGGGAGTGAAACACCCGCCATGATTCCGTAGCCGCG
CGCGAAGATACGAAGCAGATATTTCACGGACATGGCGGAGATACTTGTTTCCCGTACTAAGGTAGTC
ATGTCGGAGACATCTGAACGACAGAGCTGGCCAAGAGAACCGACCAGTTGCCCCAGGACGATCTA
GACAAAAAAAAAGAGAGATGAGTGGGCCACTTTTGCCACAACATCGACGGCCCTGCGACCGCCCC
CAGGCAAACAAACAAACCGCCGAACAATAATACTTTTGTCATTTTAGGAGGAGCGTTGTATGGATAA
AAACAACATCTCGTTGCTGCAGAATGTGGACTTCAAACTTGCAGAAAATGGGAGGCGGATTTGCAT
GATCGGAGGGTAGTTGACTCACGCCGCAGGCTGCAAATCCGTCCTCCATTATTCCATGAACAACTTC
GTAAGGTTGGGCTGAGCGCCAATGCCTAACGGACCGGGGGCCACAGCGCAACGTCCCACTTAAAG
GCCAGCGTGACATGCCAGTTCCATACCAAGTAGTGGCACCAGAGGCGGCCAATGCTCAGTAAGGGC
AGGGAGGGAGGCTCAAACGATTGGCAAAAAGAGGGGCTTGCCAGTTCAGTTCCCTGTGCGAGCGC
GAGAGGGGCAGTTTCAAATCTGGAGGGGTGTGTTGCGCTGGTCTGAAGAGAAAGAGAAGACTGTA
CTTAATAATTGTTCAAAGAGTCCATCATCGCGTTGCGGACTCCTCTAGCTGTATTTAGAGCCCTATCAT
TACTTGTCGGGTGCGAATCAAAATACCGGGATGCAGCCCTCTGGCGATTTGCATGCGGTTGTGGAGG
AAGTGAAGCCTGAATCGCGGGGCTGGGCGGCAAAGCACGACGTGAAATTCCTGGCGAAATTCGAG
GGCTTGCCCCACCGTGGTTGAAGTTTTTGTGCTGCGTAACCCCACCAACCCGCCTTGCCCCTCCCGC
CTGCCCATAAAAACTTCGACCCCTCCTCAAATCTTCTTCGATTCTTCCTCTTCACTTCCTTCGTCGGC
ATACCTGATTCAAGCAATCACCTGCCACTTTCAAGTGCGTATACCATCATCGATACACTGGTTCTTGA
CAAGTACATCGTCTCTAACTTTCCTTTTTGCAGTTTTCATTAAGCGCAAGTCGCCAGTTTCGTTCTTC
AGAATGGACTACAAGGACCATGATGGCGATTACAAGGACCACGACATCGATTATAAGGATGATGA
TGACAAGCCTCCGAGGAAACGTGCCAAAACAGAAGATGAGATGGATAAGAAGTACTCCATCGGCC
TCGACATCGGCACCAACTCCGTCGGCTGGGCCGTCATCACCGATGAGTACAAGGTCCCTTCCAAGA
AGTTCAAGGTCCTCGGCAACACCGATCGCCATTCCATCAAGAAGAACCTGATCGGCGCCCTCCTGT
TCGATTCCGGCGAAACCGCCGAGGCCACCCGCCTTAAACGCACCGCCCGTCGCCGCTACACCCGCC
GCAAGAACCGCATCTGCTACCTCCAAGAAATCTTCTCCAACGAGATGGCCAAGGTCGATGATAGC
TTCTTCCACCGCCTCGAAGAGTCCTTCCTGGTCGAAGAGGATAAGAAGCACGAGCGCCATCCTATC
TTCGGCAACATCGTCGATGAGGTCGCCTACCATGAGAAGTACCCTACCATCTACCATCTCCGCAAG
AAGCTCGTCGATTCCACCGATAAGGCCGATCTCCGCCTCATCTACCTCGCCCTCGCCCATATGATC
AAGTTCCGCGGCCATTTCCTCATCGAGGGCGATCTCAACCCTGATAACTCCGATGTCGATAAGCTG
TTCATCCAGCTCGTCCAGACCTACAACCAGCTGTTCGAGGAAAACCCTATCAACGCCTCCGGCGTC
GATGCCAAGGCCATCCTCTCCGCTCGCCTCTCCAAGTCTCGCCGCCTTGAGAACCTTATCGCCCAG

CTCCCTGGCGAGAAGAAGAACGGCCTCTTCGGCAACCTGATCGCCCTCTCCCTCGGCCTCACCCCT
AACTTCAAGTCCAACTTCGATCTCGCCGAGGATGCCAAGCTCCAGCTCTCCAAGGATACCTACGAT
GATGATCTCGATAACCTCCTCGCCCAGATCGGCGATCAGTACGCCGATCTGTTCCTCGCCGCCAAG
AACCTCTCCGATGCCATCCTCCTCTCCGACATCCTCCGCGTCAACACCGAGATCACCAAGGCCCCT
CTGTCCGCCTCCATGATCAAGCGCTACGATGAGCATCATCAGGACCTCACCCTGCTCAAGGCCCTC
GTCCGCCAGCAGCTCCCTGAGAAGTACAAAGAGATTTTCTTCGATCAGTCCAAGAACGGCTACGCC
GGCTACATCGATGGCGGCGCTTCCCAAGAAGAGTTCTACAAGTTCATCAAGCCTATCCTTGAGAAG
ATGGATGGCACCGAGGAACTCCTCGTCAAGCTCAACCGCGAGGACCTCCTCCGCAAGCAGCGCAC
CTTCGATAACGGCTCCATCCCTCATCAAATCCATCTCGGCGAGCTGCATGCCATCTTGCGCCGCCA
AGAGGATTTCTACCCATTCCTCAAGGATAACCGCGAGAAGATCGAAAAGATTCTCACCTTCCGCAT
CCCTTACTACGTCGGCCCTCTCGCTCGCGGCAACTCCCGCTTCGCCTGGATGACCCGCAAGTCCGA
GGAAACCATCACCCCTTGGAACTTCGAGGAAGTCGTCGATAAGGGCGCCTCCGCCCAGTCCTTCAT
CGAGCGCATGACCAACTTCGATAAGAACCTCCCTAACGAGAAGGTCCTCCCTAAGCACTCCCTGCT
CTACGAGTACTTCACCGTCTACAACGAGCTGACCAAGGTCAAGTACGTCACCGAGGGTATGCGCA
AGCCTGCCTTCCTGTCCGGCGAGCAGAAGAAGGCCATCGTCGATCTGCTGTTCAAGACCAACCGCA
AGGTCACCGTCAAGCAGCTCAAAGAGGATTACTTCAAGAAAATCGAGTGCTTCGATTCCGTCGAG
ATCAGCGGCGTCGAGGACCGCTTCAACGCCTCCCTCGGAACCTACCATGATCTCCTCAAGATTATC
AAGGATAAGGATTTCCTCGACAACGAGGAAAACGAGGACATCCTTGAGGACATCGTCCTCACCCT
CACCCTCTTCGAGGACCGCGAAATGATCGAGGAACGCCTCAAGACCTACGCCCATCTCTTCGATGA
TAAGGTCATGAAGCAGCTCAAGCGCCGTCGCTACACCGGCTGGGGTCGCCTCTCCCGCAAGCTCAT
CAACGGCATCCGCGATAAGCAGTCCGGCAAGACTATCCTCGATTTCCTCAAGTCCGATGGCTTCGC
CAACCGCAACTTCATGCAGCTCATCCATGATGATTCCCTCACCTTCAAAGAGGACATCCAGAAGGC
CCAGGTCAGCGGCCAGGGCGATTCCCTCCATGAGCATATCGCCAACCTCGCCGGCTCCCCTGCCAT
CAAGAAGGGCATCCTCCAGACCGTCAAGGTCGTCGATGAGCTGGTCAAGGTCATGGGCCGCCATA
AGCCTGAGAACATCGTCATCGAGATGGCCCGCGAGAACCAGACCACCCAGAAGGGCCAGAAGAA
CTCCCGCGAGCGCATGAAGCGCATCGAGGAAGGCATCAAAGAGCTGGGCAGCCAAATCCTCAAAG
AGCATCCTGTCGAGAACACCCAGCTCCAGAACGAGAAGCTCTACCTCTACTACCTCCAGAACGGC
CGCGATATGTACGTCGATCAAGAGCTGGACATCAACCGCCTCTCCGATTACGATGTCGATCATATC
GTCCCTCAGTCCTTCCTGAAGGATGATTCCATCGATAACAAGGTCCTCACCCGCTCCGATAAGAAC
CGCGGCAAGTCCGATAACGTCCCTTCCGAAGAGGTCGTCAAGAAGATGAAGAACTACTGGCGCCA
GCTCCTCAACGCCAAGCTCATCACCCAGCGCAAGTTCGATAACCTCACCAAGGCCGAGCGCGGTG
GCCTCTCCGAGCTGGATAAGGCCGGCTTCATCAAGCGCCAGCTCGTCGAAACCCGCCAGATCACC
AAGCACGTCGCCCAAATCCTCGATTCCCGCATGAACACCAAGTACGATGAGAACGATAAGCTCAT
CCGCGAAGTCAAGGTCATCACCCTCAAGTCCAAGCTCGTCAGCGATTTCCGCAAGGATTTCCAGTT
CTACAAGGTCCGCGAGATCAACAACTACCATCATGCCCATGATGCCTACCTCAACGCCGTCGTCGG
CACCGCCCTCATCAAGAAGTACCCCAAGCTCGAATCCGAGTTCGTCTACGGTGATTACAAGGTCTA
CGATGTCCGCAAGATGATCGCCAAGTCCGAGCAAGAGATCGGCAAGGCTACCGCCAAGTACTTCT
TCTACTCCAACATCATGAATTTCTTCAAGACCGAAATCACCCTCGCCAACGGCGAAATCCGCAAGC
GCCCTCTCATCGAGACTAACGGCGAGACTGGCGAGATCGTCTGGGATAAGGGCCGCGATTTCGCC
ACCGTCCGCAAGGTCCTCTCCATGCCTCAGGTCAACATCGTCAAGAAAACCGAGGTCCAGACCGG
CGGCTTCTCCAAAGAGTCCATCCTCCCCAAGCGCAACTCCGATAAGCTGATCGCCCGCAAGAAGG
ATTGGGACCCTAAGAAGTACGGCGGCTTCGATTCCCCTACCGTCGCCTACTCCGTCCTCGTCGTCG
CCAAGGTCGAGAAGGGCAAGTCCAAGAAGCTCAAGTCCGTCAAAGAGCTGCTCGGCATCACTATT
ATGGAACGCTCCAGCTTCGAGAAGAACCCTATCGATTTCCTTGAGGCCAAGGGCTACAAAGAGGT

CAAGAAGGACCTCATCATCAAGCTCCCCAAGTACTCCCTGTTCGAGCTTGAGAACGGCCGCAAGC
GCATGCTCGCCTCCGCCGGTGAGCTTCAGAAGGGCAACGAGCTGGCCCTGCCTTCCAAGTACGTCA
ACTTCCTCTACCTCGCCTCCCATTACGAGAAGCTCAAGGGCTCCCCTGAGGATAACGAGCAGAAGC
AGCTGTTCGTCGAGCAGCATAAGCACTACCTCGATGAGATCATCGAGCAGATCAGCGAGTTCTCCA
AGCGCGTCATCCTCGCCGATGCCAACCTCGATAAGGTCCTGTCCGCCTACAACAAGCACCGCGATA
AGCCTATCCGCGAGCAGGCCGAGAACATCATCCATCTCTTCACCCTCACCAACCTCGGTGCCCCTG
CCGCCTTCAAGTACTTCGATACCACCATCGATCGCAAGCGCTACACCTCCACCAAAGAGGTCCTGG
ACGCCACCCTCATCCATCAGTCCATCACCGGCCTCTACGAAACCCGCATCGATCTCTCCCAGCTCG
GCGGCGACATGGTGAGCAAGGGCGAGGAGCTGTTCACCGGGGTGGTGCCCATCCTGGTCGAGCTGGACGGCGAC
GTAAACGGCCACAAGTTCAGCGTGTCCGGCGAGGGCGAGGGCGATGCCACCTACGGCAAGCTGACCCTGAAGTTCA
TCTGCACCACCGGCAAGCTGCCCGTGCCCTGGCCCACCCTCGTGACCACCCTGACCTACGGCGTGCAGTGCTTCAGCC
GCTACCCCGACCACATGAAGCAGCACGACTTCTTCAAGTCCGCCATGCCCGAAGGCTACGTCCAGGAGCGCACCATCT
TCTTCAAGGACGACGGCAACTACAAGACCCGCGCCGAGGTGAAGTTCGAGGGCGACACCCTGGTGAACCGCATCGA
GCTGAAGGGCATCGACTTCAAGGAGGACGGCAACATCCTGGGGCACAAGCTGGAGTACAACTACAACAGCCACAAC
GTCTATATCATGGCCGACAAGCAGAAGAACGGCATCAAGGTGAACTTCAAGATCCGCCACAACATCGAGGACGGCAG
CGTGCAGCTCGCCGACCACTACCAGCAGAACACCCCCATCGGCGACGGCCCCGTGCTGCTGCCCGACAACCACTACC
TGAGCACCCAGTCCGCCCTGAGCAAAGACCCCAACGAGAAGCGCGATCACATGGTCCTGCTGGAGTTCGTGACCGCC
GCCGGGATCACTCTCGGCATGGACGAGCTGTACAAGCCTCCGAGGAAACGTGCCAAAACAGAAGATGAGT
GATGAGGATCCACTTAACGTTACTGAAATCATCAAACAGCTTGACGAATCTGGATATAAGATCGTTG
GTGTCGATGTCAGCTCCGGAGTTGAGACAAATGGTGTTCAGGATCTCGATAAGATACGTTCATTTGT
CCAAGCAGCAAAGAGTGCCTTCTAGTGATTTAATAGCTCCATGTCAACAAGAATAAAACGCGTTTTC
GGGTTTACCTCTTCCAGATACAGCTCATCTGCAATGCATTAATGCATTGACTGCAACCTAGTAACGCC
TTACAGGCTCCGGCGAAGAGAAGAATAGCTTAGCAGAGCTATTTTCATTTTCGGGAGACGAGATCA
AGCAGATCAACGGTCGTCAAGAGACCTACGAGACTGAGGAATCCGCTCTTGGCTCCACGCGACTAT
ATATTTGTCTCTAATTGTACTTTGACATGCTCCTCTTCTTTACTCTGATAGCTTGACTATGAAAATTCCG
TCACCAGCNCCTGGGTTCGCAAAGATAATTGCATGTTTCTTCCTTGAACTCTCAAGCCTACAGGACA
CACATTCATCGTAGGTATAAACCTCGAAATCANTTCCTACTAAGATGGTATACAATAGTAACCATGCA
TGGTTGCCTAGTGAATGCTCCGTAACACCCAATACGCCGGCCGAAACTTTTTTACAACTCTCCTATG
AGTCGTTTACCCAGAATGCACAGGTACACTTGTTTAGAGG
