# Supplementary material for: Development of a genome-editing CRISPR/Cas9 system in thermophilic fungal Myceliophthora species and its application to hyper-cellulase production strain engineering
Source: Biotechnol Biofuels. 2017 Jan 3;10:1. doi: 10.1186/s13068-016-0693-9 (PMC5209885; doi:10.1186/s13068-016-0693-9)
Supplement: Supplementary file 9 — Additional file 9. Nucleotide sequences of sgRNA expression cassettes. [file 13068_2016_693_MOESM9_ESM.pdf]

Additional file 3: Nucleotide sequence of the sgRNA expression cassettes. Blue letters indicate the U6 promoter. Orange letters indicate the RNA polymerase III U6 snRNA gene. Red letters indicate the sgRNA scaffold. Green letters indicate the target sequence of *amdS*, *cre-1*, *res-1*, *gh1-1*, or *alp-1*, respectively.

>RNA polymerase III U6 snRNA gene

AGGATCGGTGGAGTGAAGTTCGGAATCGAGGTTCGGCGATGGGTCGTAAGCATGGCGACTTCGAACTTACTTGC
ACTGGCAAGCGTTGCCAGAACGGCGAGAAAAAGAAGGGTAAGCGATATTCGCGTCATGATGGACTGTTCCTTT
TGGAACAGTAGTTGTTGTGGGAAGACTATGTCACACTTGCCCACCTGCAAGGCCAGGGTCGTGGTCGAACGAG
ACCAGCCTCGGCGCTGCTGGGAGCTCAAGATGGGCACGTTTGATTCGTTAGACGTCAACAAGGCTGGAGTTCC
TAGTGACAGCCAAAGGCACAGCCACATTAAGTGGCGCTTTATCTGTCCACTAAGGTTCAATTGTGGCTTTGAGC
CGCGCAGTGTGCAGTCGTGCATTGGCCACCTAGCTAGCAGTATTTAAGATCCTCTTCTCTCCCGAGATCTTCCTC
CTCTTCTTTTCTTTCTTTCCTCGCCCTTCGGGGCATTTGGTCAATTTGAAACGATACAGAGAAGATTAGCA
TGGCCCCTGCACTAAGGATGACACGCTACTCAAAGAGACGCTACCAATTTTTTT

>sgRNA-*amdS* expression cassette with U6 promoter

AGGATCGGTGGAGTGAAGTTCGGAATCGAGGTTCGGCGATGGGTCGTAAGCATGGCGACTTCGAACTTACTTGC
ACTGGCAAGCGTTGCCAGAACGGCGAGAAAAAGAAGGGTAAGCGATATTCGCGTCATGATGGACTGTTCCTTT
TGGAACAGTAGTTGTTGTGGGAAGACTATGTCACACTTGCCCACCTGCAAGGCCAGGGTCGTGGTCGAACGAG
ACCAGCCTCGGCGCTGCTGGGAGCTCAAGATGGGCACGTTTGATTCGTTAGACGTCAACAAGGCTGGAGTTCC
TAGTGACAGCCAAAGGCACAGCCACATTAAGTGGCGCTTTATCTGTCCACTAAGGTTCAATTGTGGCTTTGAGC
CGCGCAGTGTGCAGTCGTGCATTGGCCACCTAGCTAGCAGTATTTAAGATCCTCTTCTCTCCCGAGATCTTCCTC
CTCTTCTTTTCTTTCTTTCCTCGGCGAACAGCATGGAGGGTCGTTTTAGAGCTAGAAATAGCAAGTTAAAATAA
GGCTAGTCCGTTATCAACTTGAAAAAGTGGCACCGAGTCGGTGCTTTTTTT

>sgRNA-*cre-1* expression cassette with U6 promoter

AGGATCGGTGGAGTGAAGTTCGGAATCGAGGTTCGGCGATGGGTCGTAAGCATGGCGACTTCGAACTTACTTGC
ACTGGCAAGCGTTGCCAGAACGGCGAGAAAAAGAAGGGTAAGCGATATTCGCGTCATGATGGACTGTTCCTTT
TGGAACAGTAGTTGTTGTGGGAAGACTATGTCACACTTGCCCACCTGCAAGGCCAGGGTCGTGGTCGAACGAG
ACCAGCCTCGGCGCTGCTGGGAGCTCAAGATGGGCACGTTTGATTCGTTAGACGTCAACAAGGCTGGAGTTCC
TAGTGACAGCCAAAGGCACAGCCACATTAAGTGGCGCTTTATCTGTCCACTAAGGTTCAATTGTGGCTTTGAGC
CGCGCAGTGTGCAGTCGTGCATTGGCCACCTAGCTAGCAGTATTTAAGATCCTCTTCTCTCCCGAGATCTTCCTC
CTCTTCTTTTCTTTCTTTCCTCGCAACGCGCAAAGTCTGCAGGTTTTAGAGCTAGAAATAGCAAGTTAAAATAA
GGCTAGTCCGTTATCAACTTGAAAAAGTGGCACCGAGTCGGTGCTTTTTTT

>sgRNA-*res-1* expression cassette with U6 promoter

AGGATCGGTGGAGTGAAGTTCGGAATCGAGGTTCGGCGATGGGTCGTAAGCATGGCGACTTCGAACTTACTTGC
ACTGGCAAGCGTTGCCAGAACGGCGAGAAAAAGAAGGGTAAGCGATATTCGCGTCATGATGGACTGTTCCTTT
TGGAACAGTAGTTGTTGTGGGAAGACTATGTCACACTTGCCCACCTGCAAGGCCAGGGTCGTGGTCGAACGAG
ACCAGCCTCGGCGCTGCTGGGAGCTCAAGATGGGCACGTTTGATTCGTTAGACGTCAACAAGGCTGGAGTTCC
TAGTGACAGCCAAAGGCACAGCCACATTAAGTGGCGCTTTATCTGTCCACTAAGGTTCAATTGTGGCTTTGAGC
CGCGCAGTGTGCAGTCGTGCATTGGCCACCTAGCTAGCAGTATTTAAGATCCTCTTCTCTCCCGAGATCTTCCTC
CTCTTCTTTTCTTTCTTTCCTCGCCCTATGAGCCCTCGTACCGTTTTAGAGCTAGAAATAGCAAGTTAAAATAAG
GCTAGTCCGTTATCAACTTGAAAAAGTGGCACCGAGTCGGTGCTTTTTTT

>sgRNA-*gh1-1* expression cassette with U6 promoter

AGGATCGGTGGAGTGAAGTTCGGAATCGAGGTTCGGCGATGGGTCGTAAGCATGGCGACTTCGAACTTACTTGC
ACTGGCAAGCGTTGCCAGAACGGCGAGAAAAAGAAGGGTAAGCGATATTCGCGTCATGATGGACTGTTCCTTT
TGGAACAGTAGTTGTTGTGGGAAGACTATGTCACACTTGCCCACCTGCAAGGCCAGGGTCGTGGTCGAACGAG
ACCAGCCTCGGCGCTGCTGGGAGCTCAAGATGGGCACGTTTGATTCGTTAGACGTCAACAAGGCTGGAGTTCC
TAGTGACAGCCAAAGGCACAGCCACATTAAGTGGCGCTTTATCTGTCCACTAAGGTTCAATTGTGGCTTTGAGC
CGCGCAGTGTGCAGTCGTGCATTGGCCACCTAGCTAGCAGTATTTAAGATCCTCTTCTCTCCCGAGATCTTCCTC
CTCTTCTTTTCTTTCTTTCCTCGACACATTCTGCGCCATCCCGTTTTAGAGCTAGAAATAGCAAGTTAAAATAAG
GCTAGTCCGTTATCAACTTGAAAAAGTGGCACCGAGTCGGTGCTTTTTTT

>sgRNA-*alp-1* expression cassette with U6 promoter

AGGATCGGTGGAGTGAAGTTCGGAATCGAGGTTCGGCGATGGGTCGTAAGCATGGCGACTTCGAACTTACTTGC
ACTGGCAAGCGTTGCCAGAACGGCGAGAAAAAGAAGGGTAAGCGATATTCGCGTCATGATGGACTGTTCCTTT
TGGAACAGTAGTTGTTGTGGGAAGACTATGTCACACTTGCCCACCTGCAAGGCCAGGGTCGTGGTCGAACGAG
ACCAGCCTCGGCGCTGCTGGGAGCTCAAGATGGGCACGTTTGATTCGTTAGACGTCAACAAGGCTGGAGTTCC
TAGTGACAGCCAAAGGCACAGCCACATTAAGTGGCGCTTTATCTGTCCACTAAGGTTCAATTGTGGCTTTGAGC
CGCGCAGTGTGCAGTCGTGCATTGGCCACCTAGCTAGCAGTATTTAAGATCCTCTTCTCTCCCGAGATCTTCCTC
CTCTTCTTTTCTTTCTTTCCTCGTCTACCGCGGCAAGTTCAGGTTTTAGAGCTAGAAATAGCAAGTTAAAATAA
GGCTAGTCCGTTATCAACTTGAAAAAGTGGCACCGAGTCGGTGCTTTTTTT
