# Supplementary material for: Development of a genome-editing CRISPR/Cas9 system in thermophilic fungal Myceliophthora species and its application to hyper-cellulase production strain engineering
Source: Biotechnol Biofuels. 2017 Jan 3;10:1. doi: 10.1186/s13068-016-0693-9 (PMC5209885; doi:10.1186/s13068-016-0693-9)
Supplement: Supplementary file 10 — Additional file 10. Nucleotide sequences of donor DNA fragments. [file 13068_2016_693_MOESM10_ESM.pdf]

Additional file 4: Nucleotide sequences of the donor DNA fragments. Blue letters indicate the 5' flanking region of *cre-1*, *res-1*, *gh1-1*, or *alp-1*, respectively. Gray letters indicate the PtrpC-neo expression cassette. Purple letters indicate the 3' flanking region of *cre-1*, *res-1*, *gh1-1*, or *alp-1*, respectively.

> donor-*cre1*

ACCGGGGTAAGGTACTCTGTAAGTTACGGTACCTCTGGAGCCGGTGCCGCACCCCACTATTT
TTTTTCTTTTTCCCCGAGATCCCCCCTGGCATCAGACCCTCCTGGTCCCCCCCCCTGTTCCCT
CCTCACTGAATCGCTGCGTTCCCGAGCCCACCTAAGCCCCCGCTGTTCCCACACCACATGC
CAGCACACCACACGCCCCTCCCCCTATGTCAGCCCGCCCGCGTCATTTAAAACGAGTGCCG
CATCCCCCCAAGCAAACTCGCCCAACTTTTGTTACTTCCCCATGCCACTTTGAGTTTCCCCA
ACATCAGCACCCCAGACCCCAGATAACAAAGTCAAAATAAAGGTTCCCATTGTCTCCCGAC
ACAGCTCAACACGCTGTGTCAGACGAGCAACCACCAAACACATATCGCTTCGATACTCGAC
CAAGAACGACGTGCCTGACATCAGTACCCCTCGTCCCACACTCGAAGTTGGGCGGCTTGTT
GTTTTTCTCCGGTTGCGTGCTCCGCGTGCAACCAAGTTGAAAGAGTCCGGGAACAACATCA
GGCTGAGATCTGGTGTTGGAGCAACTCCGCTTGGGAGGGTGGACATACGACGTTAACTGAT
ATTGAAGGAGCATTTTTTGGGCTTGGCTGGAGCTAGTGGAGGTCAACAATGAATGCCTATTT
TGGTTTAGTCGTCCAGGCGGTGAGCACAAAATTTGTGTCGTTTGACAAGATGGTTCATTTAG
GCAACTGGTCAGATCAGCCCCACTTGTAGCAGTAGCGGCGGCGCTCGAAGTGTGACTCTTA
TTAGCAGACAGGAACGAGGACATTATTATCATCTGCTGCTTGGTGCACGATAACTTGGTGCG
TTTGTCAAGCAAGGTAAGTGGACGACCCGGTCATACCTTCTTAAGTTCGCCCTTCCTCCCTT
TATTTCAGATTCAATCTGACTTACCTATTCTACCCAAGCATCCAAATGATTGAACAAGATGGA
TTGCACGCAGGTTCTCCGGCCGCTTGGGTGGAGAGGCTATTCGGCTATGACTGGGCACAAC
AGACAATCGGCTGCTCTGATGCCGCCGTGTTCCGGCTGTCAGCGCAGGGGCGCCCGGTTCT
TTTTGTCAAGACCGACCTGTCCGGTGCCCTGAATGAACTGCAAGACGAGGCAGCGCGGCT
ATCGTGGCTGGCCACGACGGGCGTTCCTTGCGCAGCTGTGCTCGACGTTGTCACTGAAGCG
GGAAGGGACTGGCTGCTATTGGGCGAAGTGCCGGGGCAGGATCTCCTGTCATCTCACCTTG
CTCCTGCCGAGAAAGTATCCATCATGGCTGATGCAATGCGGCGGCTGCATACGCTTGATCCG
GCTACCTGCCCATTCGACCACCAAGCGAAACATCGCATCGAGCGAGCACGTACTCGGATGG
AAGCCGGTCTTGTCGATCAGGATGATCTGGACGAAGAGCATCAGGGGCTCGCGCCAGCCG
AACTGTTCGCCAGGCTCAAGGCGAGCATGCCCGACGGCGAGGATCTCGTCGTGACCCATG
GCGATGCCTGCTTGCCGAATATCATGGTGGAAAATGGCCGCTTTTCTGGATTCATCGACTGT
GGCCGGCTGGGTGTGGCGGACCGCTATCAGGACATAGCGTTGGCTACCCGTGATATTGCTG
AAGAGCTTGGCGGCGAATGGGCTGACCGCTTCCTCGTGCTTTACGGTATCGCCGCTCCCGA
TTCGCAGCGCATCGCCTTCTATCGCCTTCTTGACGAGTTCTTCTGAAGTTCCCCGGGTGTAG
CAAACGCTTCTCGAGGTCCGACGAGCTCACCCGGCACTCGAGGATACACAACAATCCCAA
CTCGCGGAGAGGAAACAAGGGCCATCATCAGCACGCCGCCATGATCCAGAGGATGCAGCA
CGACGCCATGGCACCGCCACCGGGGCCCAAGATGATCCGCTCGGCGCCCCCCACGGCATTG
TCTTCACCCAACGTGTCGCCTCCTCACTACAGCTCCTATCCCATGAATCTTCCCCCGCCGCT
CAGTCTCGGCCCGTACAACCGCGGTGCCTTGAGCAGCCAGAGTGGTTCGGATGTGGCAATG
TTGGCGAGGACGGCCGACCGGGACACCACCCCGAGTCAGTCTCATTACCCGCCGTCGAGA
CAGCACCCGTACTACGGCGGGTCGCTCCACTCGTCGCGGAACCCGCTGCCCGGTCTGGGCG
CCTACCACATGTCGCGGTCGCACTCGAACGATGACCACGACGACCACTATGCGCAAAGTTA
CAGGCAGCCGAAGAGGTCGAGGCCCAACTCCCCCAACTCGACGGCTCCTTCCTCTCCTACC

TTTTCCCACAACTCGTTGTCCCCGACGCCGGACCACACCCCA

> donor-*res-1*

AACCAACCCTCCTTCGACTTTCCGGCCGCCAAAGCGCCAGGATTCGTTGGTGAGTCGTCAC
AGATCCCTAGGTCCTCTCCTTCTCGTCCTTCTCCGCCTCCGCCCAGTCACGGCGGACCAGCC
CGCCGTGGCTCTCTCTCTTCTTCCGAGTCTTGCGCTTCCGAGTCCACGGTCGTCGCCGCCCC
CAAGCCCTCCAGTCCCGGCCTGGCGGTCGACACACGCCTTGCGCGGCAGACGACCACCAT
GTCTGCATCCCTGGCGTCCACCCCTGCCTCTTCCTCTTCTGGCCTCCTCGTGACGCCGCCCA
CGACCTCTGCTTCTTCCTTTTCTTCTTCTTCTTCTTCTTTTTCTTCTTCTTCCTCTTCTCTC
GCCGCCTGGAGCAGCCCGCCCGCGGCCTGCGGCTCGCCCTCGCGCACGGGCACCCCCAAC
GCCCGCATGCTCTCTTCTTTCTTTTTTCAATCTTCTGGGCATTTCGTCCCGCCTCTCGAGACC
TGTTGGTTTCCTCTCTCGCGATTGCGAGATCCCACCGCACGACCGGGTTTTGCTGACATGAA
AAACCCTCGCGCCTACAGATCCCTCCTTAATTCACCCGGACGTCGACGTTAACTGATATTGA
AGGAGCATTTTTTGGGCTTGGCTGGAGCTAGTGGAGGTCAACAATGAATGCCTATTTTGGTT
TAGTCGTCCAGGCGGTGAGCACAAAATTTGTGTCGTTTGACAAGATGGTTCATTTAGGCAA
CTGGTCAGATCAGCCCCACTTGTAGCAGTAGCGGCGGCGCTCGAAGTGTGACTCTTATTAG
CAGACAGGAACGAGGACATTATTATCATCTGCTGCTTGGTGCACGATAACTTGGTGCGTTTG
TCAAGCAAGGTAAGTGGACGACCCGGTCATACCTTCTTAAGTTCGCCCTTCCTCCCTTTATT
TCAGATTCAATCTGACTTACCTATTCTACCCAAGCATCCAAATGATTGAACAAGATGGATTG
CACGCAGGTTCTCCGGCCGCTTGGGTGGAGAGGCTATTCGGCTATGACTGGGCACAACAGA
CAATCGGCTGCTCTGATGCCGCCGTGTTCCGGCTGTCAGCGCAGGGGCGCCCGGTTCTTTT
TGTCAAGACCGACCTGTCCGGTGCCCTGAATGAACTGCAAGACGAGGCAGCGCGGCTATC
GTGGCTGGCCACGACGGGCGTTCCTTGCGCAGCTGTGCTCGACGTTGTCACTGAAGCGGG
AAGGGACTGGCTGCTATTGGGCGAAGTGCCGGGGCAGGATCTCCTGTCATCTCACCTTGCT
CCTGCCGAGAAAGTATCCATCATGGCTGATGCAATGCGGCGGCTGCATACGCTTGATCCGGC
TACCTGCCCATTCGACCACCAAGCGAAACATCGCATCGAGCGAGCACGTACTCGGATGGAA
GCCGGTCTTGTCGATCAGGATGATCTGGACGAAGAGCATCAGGGGCTCGCGCCAGCCGAA
CTGTTCGCCAGGCTCAAGGCGAGCATGCCCGACGGCGAGGATCTCGTCGTGACCCATGGCG
ATGCCTGCTTGCCGAATATCATGGTGGAAAATGGCCGCTTTTCTGGATTCATCGACTGTGGC
CGGCTGGGTGTGGCGGACCGCTATCAGGACATAGCGTTGGCTACCCGTGATATTGCTGAAG
AGCTTGGCGGCGAATGGGCTGACCGCTTCCTCGTGCTTTACGGTATCGCCGCTCCCGATTCG
CAGCGCATCGCCTTCTATCGCCTTCTTGACGAGTTCTTCTGAGTCTTGTCTCGTACCAGTCC
CACTTCTCTGGCGAGCAGCCATTCGGTGGCGACGAGTCGAGGGACAAGCAGCGCTGCCCG
CACCAGGACTGCGGCAAGACCTTCAAGGACCTCAAGGCTCATATGCTCACCCACCAGAAC
GAGCGGCCCGAGAAGTGCCCCATCACGACGTGCGAGTATCACGTAAAAGGCTTCGCGAGA
AAGTGAGTAGTAATATAGTAGTAGCCTGTGTGCCCGGCTCTTCAAGAACCCCTGAACACAA
CAACAAACACAAGGCTCAGAGCGCTAACCGTGCCAATCTTGATCAGGTACGACAAGAACC
GCCACACGCTCACCCACTACAAGGGCACCATGGTGTGCGGCTTCTGCCCGGGGTCGGGCTC
CGCGGCGGAGAAGTCGTTCAACCGGGCCGATGTGTTCAAGAGGCACTTGACGGCCGTGCA
CGGAGTCGAGCAGACGCCTCCCAACGGTCGGAAGAAGACGGCCGGCGGCAGCAGCAGCG
GGAACGACGCGAAGCTCACGGGCTATGCGCCCGACGCGACGGGCAAGTGCTCGACGTGCT
CGCAAACTTTCAGCAACGCACAAGACTTTTACGAGCACC

> donor-*gh1-1*

ACTATCAACAGCCCCCCGGATTGCTGACCGTTGGCCATTCACGGCCCTTCGTTTGCCAGTCC
TTCGCCAGGGTCAACCTACCCCGCGGTGGGGGGTTGTTCTTGGATCCTTGTCGAGGCCCCG
GCTGCCCACATCGCCCACGCTGTGCACTCAGCGTAACACAGGGCCCGGATCTCTCAAGCGA
TGCCCAGCTTTTTTTCATCGGTGTTGACGGTCCGAAACTCGCGGGAGAGATGGGGGCAGAT
CATGGCGGGAAACGGCCGTGATGGTTCCTGGATATAAAGGAGATCAGGCCTTCCCTCCTCG
GCTCATTGGGGCCTACTAGCACATCATCATCCGTCTTCCATCCCTCCTCAGAACTTCCTTCCC
CTTCCTCCTATCCACCTTTCCCTTACTCACACAGACAATCGTCCATCGTCCACCATGTCTCTT
CCCAAGGACTTCAAGTGGGGCTTCGCCACCGCCTCGTAAGTTCAAGGACCCGGGCTTTTCG
ATCAAGCTCACAGAACCGTCCTTGGCTGACTGTGTGTTCCCTTTCTCTCCTCACCCACAGGT
ACCAGATTGAGGGCTCCGTCAACGAGGATGGCCGTGGCCCCTCCACGACGTTAACTGATAT
TGAAGGAGCATTTTTTGGGCTTGGCTGGAGCTAGTGGAGGTCAACAATGAATGCCTATTTTG
GTTTAGTCGTCCAGGCGGTGAGCACAAAATTTGTGTCGTTTGACAAGATGGTTCATTTAGGC
AACTGGTCAGATCAGCCCCACTTGTAGCAGTAGCGGCGGCGCTCGAAGTGTGACTCTTATT
AGCAGACAGGAACGAGGACATTATTATCATCTGCTGCTTGGTGCACGATAACTTGGTGCGTT
TGTCAAGCAAGGTAAGTGGACGACCCGGTCATACCTTCTTAAGTTCGCCCTTCCTCCCTTTA
TTTCAGATTCAATCTGACTTACCTATTCTACCCAAGCATCCAAATGATTGAACAAGATGGATT
GCACGCAGGTTCTCCGGCCGCTTGGGTGGAGAGGCTATTCGGCTATGACTGGGCACAACAG
ACAATCGGCTGCTCTGATGCCGCCGTGTTCCGGCTGTCAGCGCAGGGGCGCCCGGTTCTTT
TTGTCAAGACCGACCTGTCCGGTGCCCTGAATGAACTGCAAGACGAGGCAGCGCGGCTAT
CGTGGCTGGCCACGACGGGCGTTCCTTGCGCAGCTGTGCTCGACGTTGTCACTGAAGCGG
GAAGGGACTGGCTGCTATTGGGCGAAGTGCCGGGGCAGGATCTCCTGTCATCTCACCTTGC
TCCTGCCGAGAAAGTATCCATCATGGCTGATGCAATGCGGCGGCTGCATACGCTTGATCCGG
CTACCTGCCCATTCGACCACCAAGCGAAACATCGCATCGAGCGAGCACGTACTCGGATGGA
AGCCGGTCTTGTCGATCAGGATGATCTGGACGAAGAGCATCAGGGGCTCGCGCCAGCCGA
ACTGTTCGCCAGGCTCAAGGCGAGCATGCCCGACGGCGAGGATCTCGTCGTGACCCATGGC
GATGCCTGCTTGCCGAATATCATGGTGGAAAATGGCCGCTTTTCTGGATTCATCGACTGTGG
CCGGCTGGGTGTGGCGGACCGCTATCAGGACATAGCGTTGGCTACCCGTGATATTGCTGAA
GAGCTTGGCGGCGAATGGGCTGACCGCTTCCTCGTGCTTTACGGTATCGCCGCTCCCGATTC
GCAGCGCATCGCCTTCTATCGCCTTCTTGACGAGTTCTTCTGATGTTCAAGGCCATCCCCAA
GTGCAAGCACTGGATCACCTTCAACGAGCCGTGGTGCACGTCCATCCTGGGCTACAACACG
GGCTACTTCGCGCCCGGCCGCACGTCGGACCGCAGCAAGTCGCCCGTCGGCGACAGCGCG
CGCGAGCCGTGGATCGTCGGCCACAACATCCTCATCGCGCACGGGAGGGCCGTCAAGGCG
TACCGCGAGGACTTCAAGCCCACGCAGGGCGGCGAGATCGGCATCACGCTCAACGGCGAC
GCCACGCTCCCCTGGGACCCGGAGGACCCGGCCGACGTCGAGGCGTGCGACCGCAAGATC
GAGTTCGCCATCTCGTGGTTCGCCGACCCCATCTACTTTGGCGAGTACCCGGCGTCGATGCG
CAAGCAGCTGGGCGACCGCCTGCCCAAGTTCACGGCCGAGGAGGTGGCGCTCGTCAAGGG
CTCCAACGACTTCTACGGCATGAACCACTACACGGCCAACTACATCAAGCACAAGAAGGG
CGTGCCGCCCGAGGACGACTTCCTGGGCAACCTCGAGACGCTCTTCTACAACAAGAACGC
CGACTGCATCGGGCCCGAGACGCAGTCCTTCTGGCTGC

> donor-*alp-1*

CCTTCCTAGTCCTCGCAGCAAACTCGAGCTGCTATCAGATTCCCGGGAAGCGGCCTGCCACAGCCGCT
CAACCCGGCCTTGGCATGGCCAGGCGGCCCTTTCATGTGTCGAAAGCGGCAGGTCATCAGCACAGATC
TCGAGGGTGGGAAAGAGAGGGGGGGGAGGGGCGATGCTGGGGCGATGCTGCTTGGAGCCGCATCCG

GGGAGGGGGCCCTGCTGTTCATCCATATCCAGGATGATGCGAGATTGAAGCAAGATAAATAACACGGC
TTCCCCCTCCCCTTTCGATCCGGACCAGACCATCGTCTCCAACACCCCAAAGTCGATCCGACAAGTCC
CAATCCACCCCGCCCGCCCCTCCCTCCGTCGCCGTCCCGGTCTTCCGATTTCGTCAAGATGCACTTCTC
CACCGCTCTCCTGGCCTTCCTGCCCGCCGCCCTCGCGGCCCCTACTGCCGAGACCCTCGACAAGCGCG
CCCCGATCCTGACTGCTCGCGCTGGCCAGGTCGTCCCGGGCAAGTACATCATCAAGCTCCGCGACGGA
GCCAGCGACGATGTCCTTGAGGCCGCCATCGGCAAGCTCCGCTCCAAGGCCGACCACCGACGTTAAC
TGATATTGAAGGAGCATTTTTTGGGCTTGGCTGGAGCTAGTGGAGGTCAACAATGAATGCCTATTTTGG
TTTAGTCGTCCAGGCGGTGAGCACAAAATTTGTGTCGTTTGACAAGATGGTTCATTTAGGCAACTGGT
CAGATCAGCCCCACTTGTAGCAGTAGCGGCGGCGCTCGAAGTGTGACTCTTATTAGCAGACAGGAACG
AGGACATTATTATCATCTGCTGCTTGGTGCACGATAACTTGGTGCGTTTGTCAAGCAAGGTAAGTGGAC
GACCCGGTCATACCTTCTTAAGTTCGCCCTTCCTCCCTTTATTTCAGATTCAATCTGACTTACCTATTCTA
CCCAAGCATCCAAATGATTGAACAAGATGGATTGCACGCAGGTTCTCCGGCCGCTTGGGTGGAGAGG
CTATTCGGCTATGACTGGGCACAACAGACAATCGGCTGCTCTGATGCCGCCGTGTTCCGGCTGTCAGC
GCAGGGGCGCCCGGTTCTTTTTGTCAAGACCGACCTGTCCGGTGCCCTGAATGAACTGCAAGACGAG
GCAGCGCGGCTATCGTGGCTGGCCACGACGGGCGTTCCTTGCGCAGCTGTGCTCGACGTTGTCACTGA
AGCGGGAAGGGACTGGCTGCTATTGGGCGAAGTGCCGGGGCAGGATCTCCTGTCATCTCACCTTGCTC
CTGCCGAGAAAGTATCCATCATGGCTGATGCAATGCGGCGGCTGCATACGCTTGATCCGGCTACCTGCC
CATTCGACCACCAAGCGAAACATCGCATCGAGCGAGCACGTACTCGGATGGAAGCCGGTCTTGTCGAT
CAGGATGATCTGGACGAAGAGCATCAGGGGCTCGCGCCAGCCGAACTGTTCGCCAGGCTCAAGGCGA
GCATGCCCGACGGCGAGGATCTCGTCGTGACCCATGGCGATGCCTGCTTGCCGAATATCATGGTGGAA
AATGGCCGCTTTTCTGGATTCATCGACTGTGGCCGGCTGGGTGTGGCGGACCGCTATCAGGACATAGC
GTTGGCTACCCGTGATATTGCTGAAGAGCTTGGCGGCGAATGGGCTGACCGCTTCCTCGTGCTTTACG
GTATCGCCGCTCCCGATTCGCAGCGCATCGCCTTCTATCGCCTTCTTGACGAGTTCTTCTGAGGCTCCA
CCACCTACACCTACGACACCAGCGCCGGCGAGGGCACCTGTGCCTATGTGATCGACACGGGCATCTAC
ACTAGCCACTCCGTATGTCTCGCGGTTACCTCCCCTTTCGGAAGAAGGGGCATCCATATGCTGACCCCT
CCTGATCACAGGACTTCGGCGGCCGTGCCACTTTCGCCGCCAACTTCGTCGACAGCTCTAACACCGAT
GGCAACGGCCACGGCACCCACGTCGCCGGCACCATCGGCGGCACCACGTACGGTGTTGCCAAGAAG
ACCAAGCTCTACGCCGTCAAGGTTCTCGGCTCCGACGGCTCTGGCACCACGTATGCCTCGCACCCGCG
CACCCGCACACCCGCCCGGCCGTTATCTTCTGACTGACATTCCTCTTTCTCCTCTCTAGTTCTGGTGTC
ATTGCTGGCATCAACTTCGTCGCTGACGACGCGCCCAAGCGCAGCTGCCCCAAGGGCGTCGTCGCCA
ACATGTCGCTCGGCGGTAGCTACTCGGCCTCCATCAACAACGCCGCCGCCGCCCTCGTCAGGTCGGGC
GTCTTCCTGGCCGTCGCCGCCGGCAACGAGAACCAGAACGCCGCCAACTC
